# A meta-analysis of the diagnostic utility of biomarkers in cerebrospinal fluid in Parkinson’s disease

**DOI:** 10.1038/s41531-022-00431-7

**Published:** 2022-11-29

**Authors:** Chunchen Xiang, Shengri Cong, Xiaoping Tan, Shuang Ma, Yang Liu, Hailong Wang, Shuyan Cong

**Affiliations:** 1 Department of Neurology, Shengjing Hospital of China Medical University, Shenyang, China; 2 Department of Neurology, Beijing Tiantan Hospital, Capital Medical University, Beijing, China; 3 Department of Clinical Epidemiology and Evidence-Based Medicine, First Hospital of China Medical University, Shenyang, China

**Keywords:** Parkinson's disease, Diagnostic markers

## Abstract

Biomarkers play important roles in the diagnosis and differential diagnosis of Parkinson’s disease (PD). Thus, we carried out a systematic review and meta-analysis evaluating the diagnostic utility of cerebrospinal fluid (CSF) biomarkers to distinguish PD from atypical parkinsonian syndromes (APSs) and controls. Data for PD and APS and controls were extracted from 123 studies that reported the concentration of CSF biomarkers. Comparisons were presented using pooled Hedges’ *g*. Sources of heterogeneity were evaluated using meta-regression, and subgroup and sensitivity analyses. We found that compared with controls, PD patients had lower levels of amyloid beta 1-42, phosphorylated tau, total tau, total α-synuclein, Zn, DJ-1, and YKL-40, and higher levels of oligomeric and phosphorylated α-synuclein. Moreover, lower CSF levels of neurofilament light chain, t-tau, YKL-40, and C-reactive protein were found in PD patients compared to those with multiple system atrophy. PD patients also had lower levels of NFL and higher levels of Aβ42 compared with patients with progressive supranuclear palsy. Reduced levels of p-tau and t-tau and higher Aβ42 levels were found in PD patients compared with patients with dementia with Lewy bodies. Finally, reduced NFL levels were found in patients with PD compared with patients with cortical basal degeneration. Therefore, we believe that the combinations of t-α-syn, Aβ42, and NFL could be promising biomarkers for the differential diagnosis of PD and APSs.

## Introduction

Parkinson’s disease (PD) is a progressive neurological disorder with increasing incidence in recent years^1^. Currently, the diagnosis of PD primarily relies on the identification of cardinal motor symptoms, including resting tremor, bradykinesia, and rigidity^2^. Unfortunately, when motor manifestations appear, nearly 70% of nigral neurons are lost^3^. Moreover, there exist considerable clinical overlaps with atypical parkinsonian syndromes (APSs) (e.g., progressive supranuclear palsy [PSP], dementia with Lewy bodies [DLB], cortical basal degeneration [CBD] and multiple system atrophy [MSA]), which are heterogeneous neurodegenerative disorders that are distinct from PD but share its central characteristic of akinetic rigidity. Thus, it is not surprising to observe the underdiagnosis of APSs and overdiagnosis of PD^4,5^. Identifying diagnostic and prognostic biomarkers of PD is important in the area of neurodegenerative disorders. Alzheimer’s disease (AD) provides a good example of the use of cerebrospinal fluid (CSF) biomarkers for its diagnosis, independent of the clinical stage^6^.

CSF is in close contact with the central nervous system and can reflect the biochemical processes occurring in the brain. Thus, CSF biomarkers can better reflect disease stages compared with blood or other biomarkers. Research on CSF biomarkers in PD has markedly expanded^7–9^; however, the available data are often controversial. Several systematic reviews on this topic have been published currently; however, each review only investigated one specific biomarker in PD^10–12^. Moreover, no meta-analyses on the diagnostic utility of potentially important biomarkers in PD (i.e., amyloid beta 1-42 [Aβ42] and tau) have been published^3,13^. Thus, we conducted a systematic review and meta-analysis evaluating the diagnostic utility of important CSF biomarkers for distinguishing PD patients from controls (primary outcome) and patients with different APSs (secondary outcome).

**Table 1:** Performance of potential CSF biomarkers for PD and parkinsonism patients and controls.

Biomarker	Pathophysiological mechanism	PD versus HC/OND	PD versus HC	PD versus OND	PD versus MSA	PD versus PSP	PD versus DLB	PD versus CBD
Aβ42	Anti-oxidative stress; protects nerves	↓↓	↓↓	↓↓	↑	↑↑	↑↑	NA
t-tau	Induces and promotes tubulin formation; stabilizes microtubules	↓↓	↓↓	↓	↓↓	↓	↓↓	NA
p-tau	Loss of neuronal dopaminergic processes	↓↓	↓↓	↓	−	↓	↓↓	NA
NFL	Important components of neuron cytoskeleton; axonal damage	NA	↑	NA	↓↓	↓↓	NA	↓↓
t-α-syn	Nerve cell synuclein deposition	↓↓	↓↓	↓↓	↑↑	↓	−	NA
p-α-syn	Nerve cell synuclein deposition	↑↑	NA	NA	NA	NA	NA	NA
o-α-syn	Nerve cell synuclein deposition	↑↑	↑↑	↑↑	NA	NA	NA	NA
DJ-1	Oxidative stress	NA	↓↓	NA	NA	NA	NA	NA
YKL-40	Neuroinflammation	NA	↓↓	NA	↓↓	NA	NA	NA
CRP	Neuroinflammation	NA	↑	NA	↓↓	NA	NA	NA
IL-6	Neuroinflammation	NA	↑	NA	NA	NA	NA	NA
FLT-3	Neuroimmunity	NA	NA	NA	↑	NA	NA	NA
Cu	Others^a^	NA	↓	NA	NA	NA	NA	NA
Fe	Others^a^	NA	↑	NA	NA	NA	NA	NA
Mn	Others^a^	NA	↓	NA	NA	NA	NA	NA
Zn	Others^a^	NA	↓↓	NA	NA	NA	NA	NA
Arginine	Dopamine metabolism	NA	↓	NA	NA	NA	NA	NA
Citrulline	Dopamine metabolism	NA	↓	NA	NA	NA	NA	NA

The meta-analysis revealed that these biomarkers were altered in PD patients relative to other neurological disorders (e.g., atypical parkinsonian syndromes). Relevant references are included in the online supplementary material. *P* < 0.05 was regarded as significant, positive or negative; ↑↑, increased and positive; ↑, increased but negative; −, no change; ↓↓, decreased and positive; ↓, decreased but negative.

*PD* Parkinson’s disease, *PSP* progressive supranuclear palsy, *MSA* multiple system atrophy, *DLB* dementia with Lewy bodies, *CBD* cortico-basal degeneration, *HC* healthy control, *OND* other neurodegenerative diseases, *Aβ42* the 42-amino-acid form of Aβ, *p-tau* phosphorylated tau, *t-tau* total tau, *NFL* neurofilament light-chain protein, *α-syn* α-synuclein, *IL-6* interleukin-6, *CRP* C-reactive protein, *YKL-40, CHI3L1* chitinase-3-like protein 1, *Cu* copper, *Mn* manganese, *Fe* iron, *Zn* zinc.

^a^Oxidative stress, glutathione depletion, glutamate neurotoxicity, alpha-synuclein oligomerization, Lewy body formation, iron homeostasis, and neurotransmitter synthesis.

## Results

### Literature search findings

**Fig. 1:**
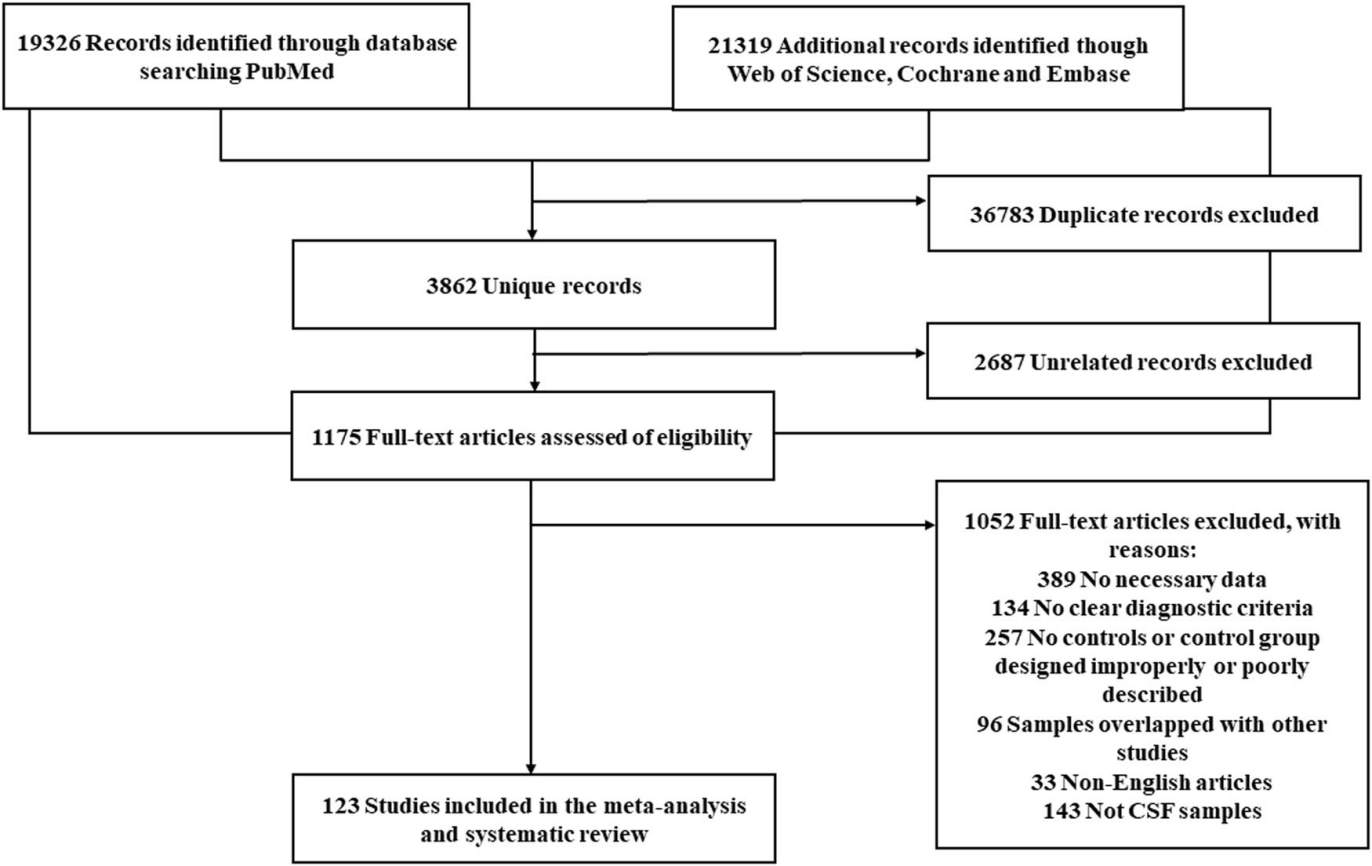
Flow chart of the systematic article selection strategy. From 19326 studies from Pubmed, and 21319 studies from Science, Cochrane and Embase identified from the search strategy, a total of 123 articles were included for review. The boxes indicate exclusions of articles during each of the following screening stages: duplicate recoeds, unrelated recos and others. Reasons for exclusion are shown inside the right boxes. The box at bottom indicates inclusion articles.

**Fig. 2:**
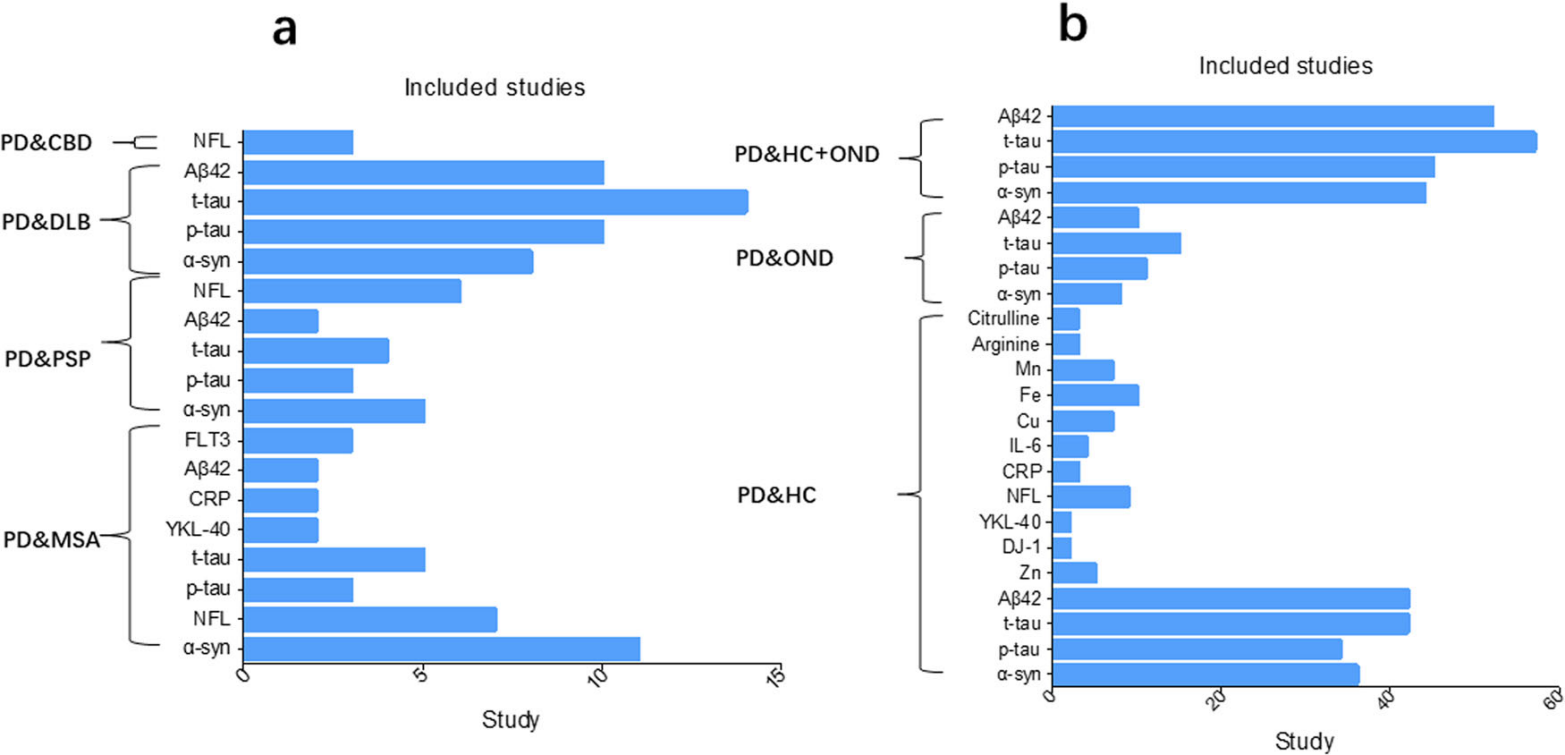
The number of studies in the meta-analysis for PD versus the parkinsonism and control groups. **a** The number of included studies for the PD versus parkinsonism groups. The number of studies included for the indicated biomarkers comparing PD patients to those with MSA, PSP, DLB, or CBD is illustrated. **b** The number of included studies for the PD versus control groups. The number of studies included for the indicated biomarkers comparing PD patients to the control group or the combined HC and OND groups is illustrated. PD Parkinson’s disease, PSP progressive supranuclear palsy, MSA multiple system atrophy, DLB dementia with Lewy bodies, CBD cortico-basal degeneration, HC healthy control, OND other neurodegenerative diseases, Aβ42 the 42-amino-acid form of Aβ, p-tau phosphorylated tau, t-tau total tau, NFL neurofilament light-chain protein, α-syn α-synuclein, IL-6 interleukin-6, CRP C-reactive protein, CHI3L1 YKL-40, chitinase-3-like protein 1, Cu copper, Mn manganese, Fe iron, Zn zinc.

**Fig. 3:**
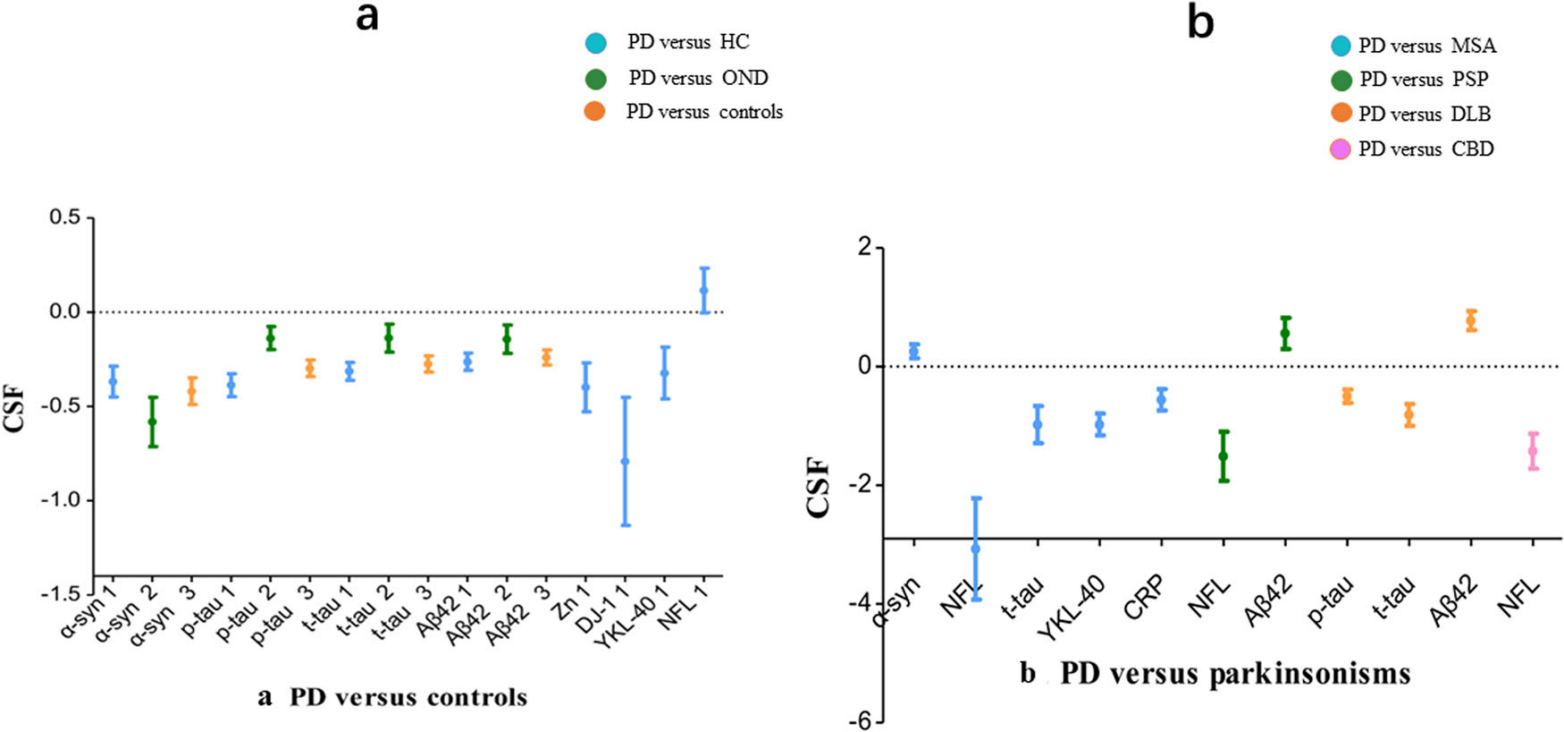
Meta-analysis for CSF biomarker performance measured with the effect size (ES) and 95% confidence interval (CI) for PD versus control or parkinsonism patients. **a** The biomarkers illustrated in blue and marked as 1 were significantly different between PD patients and HC. The biomarkers illustrated in green and marked as 2 were significantly different between PD and OND patients. A significant difference was identified between the PD patients and those in the OND and HC groups combined for the orange-labeled biomarkers marked as 3. **b** The biomarkers illustrated in blue were significantly different between PD and MSA patients. The biomarkers illustrated in green were significantly different between PD and PSP patients. A significant difference was observed between the PD and DLB patients for the orange-labeled biomarkers. The biomarker marked pink was significantly different between the PD and CBD patients. PD Parkinson’s disease, OND other neurodegenerative diseases, HC healthy control, MSA multiple system atrophy, DLB dementia with Lewy bodies, PSP progressive supranuclear palsy, CBD cortico-basal degeneration, α-syn α-synuclein, Aβ42 the 42-amino-acid form of Aβ, t-tau total tau, p-tau phosphorylated tau, NFL neurofilament light-chain protein, YKL-40, CHI3L1 chitinase-3-like protein 1.

We identified 3862 articles after duplicate search removal (Fig. 1). Among these studies, 123 focused on CSF biomarkers and were included in the analysis. Overall, our analysis consisted of 11688 PD patients, 6735 controls (5757 healthy controls [HC], 978 other neurology disorders [OND]), 859 MSA patients, 327 PSP patients, 708 DLB patients, and 32 patients with CBD. The quality assessment and baseline characteristics of our study are reported in Supplementary Fig. 3. Quantitative analysis results for 16 biomarkers from our meta-analysis are provided in Supplementary Table 4. Figures 2 and 3 show the results with *P* < 0.05. The remaining results are presented in Supplementary Table 5. Forest plots for those biomarkers are available in Supplementary Figs. 6–10. The results of performance of potential CSF biomarkers for PD and parkinsonism patients and controls were listed in Table 1.

### PD patients versus controls

A total of 103 studies (9192 PD patients, 5661 controls) assessed the performance of Aβ42, p-tau, t-tau, t-α-syn, p-α-syn, o-α-syn, NFL, Zn, YKL-40, CRP, IL-6, Cu, Fe, Mn, arginine, DJ-1, and citrulline in distinguishing PD patients from controls. Random-effect results showed that PD patients had lower CSF levels for Aβ42 (SMD = −0.239, 95% confidence interval [CI]: −0.309 to −0169), p-tau (SMD = −0.302, 95% CI: −0.376 to −0.228), t-tau (SMD = −0.274, 95% CI: −0.349 to −0.200), t-α-syn (SMD = −0.419, 95% CI: −0.542 to −0.295), Zn (SMD = −0.398, 95% CI: −0.623 to −0.173), DJ-1 (SMD = −0.791, 95% CI: −1.380 to −0.202), and YKL-40 (SMD = −0.322, 95% CI: −0.561 to −0.082) (Fig. 3). In contrast, the CSF levels of o-α-syn (SMD = 1.754, 95% CI: 0.590 to 2.919) and phosphorylated α-syn (p-α-syn) were elevated. No significant differences were found in NFL, CRP, IL-6, Cu, Fe, Mn, arginine, or citrulline concentrations between PD patients and controls.

A high level of heterogeneity was found for DJ-1, and the sensitivity analysis showed that the results for the performance of most CSF biomarkers were not affected by specific studies. Significant bias was not found in most studies, as indicated by Egger’s test and funnel plots. Meta-regression and subgroup analysis were performed to potentially identify the primary source of the heterogeneity. A significant difference was not observed between the subgroup analysis and overall results. However, the random-effect results showed that no significant differences were found for t-tau (SMD = −0.129, 95% CI: −0.269 to 0.012) or p-tau (SMD = −0.13, 95% CI: −0.27 to 0.01) when ONDs were used as controls.

Therefore, DJ-1, t-α-syn, Zn, and YKL-40 significantly distinguished PD from controls with a moderate effect size. However, because of the limited number of studies included in the analysis for DJ-1, Zn, and YKL-40, more studies are needed to further identify their potential diagnostic values. Nevertheless, we believe that t-α-syn will help distinguish PD from controls.

### PD versus atypical parkinsonian syndromes

#### PD versus MSA

Seventeen studies (1,383 PD patients, 503 MSA patients) assessed the capacity of NFL, t-α-syn, t-tau, YKL-40, p-tau, FLT-3, and CRP in differentiating PD and MSA patients. Lower CSF levels of NFL (SMD = −3.609, 95% CI: −4.545 to −1.594), t-tau (SMD = −0.977, 95% CI: −1.520 to −0.434), YKL-40 (SMD = −0.973, 95% CI: −1.292 to −0.655), and CRP (SMD = −0.556, 95% CI: −0.871 to −0.242) were observed in PD patients compared with MSA patients. In contrast, the CSF levels of t-α-syn (SMD = 0.257, 95% CI: 0.055 to 0.459) were elevated. No significant differences were detected for p-tau, Aβ42, or FLT-3 between these two groups.

Therefore, NFL and YKL-40 can significantly distinguish PD from MSA with a large effect size, while t-tau and CRP can only distinguish with a moderate effect size. Because of the small number of studies included for YKL-40 and CRP, they cannot be considered promising biomarkers at this time.

#### PD Versus PSP

Ten studies (1243 PD patients, 327 PSP patients) assessed the performance of NFL, Aβ42, t-α-syn, and p-tau in distinguishing between PD and PSP patients. Lower CSF levels of NFL (SMD = −1.509, 95% CI: −2.222 to −0.796) and higher Aβ42 levels were observed in PD patients compared with the PSP patients (SMD = 0.561, 95% CI: 0.103 to 1.018). However, no significant differences were detected for t-α-syn or p-tau between these two groups.

In general, NFL can significantly distinguish PD from PSP with a large effect size, while Aβ42 could only distinguish with a moderate effect size.

#### PD versus DLB

Twenty studies (1,384 PD patients, 708 DLB patients) assessed the performance of p-tau, t-tau, t-α-syn, and Aβ42 in distinguishing PD from DLB. Reduced CSF levels of p-tau (SMD = −0.495, 95% CI: −0.689 to −0.301) and t-tau (SMD = −0.626, 95% CI: −0.888 to −0.365) and higher Aβ42 levels (SMD = 0.775, 95% CI: 0.498 to 1.052) were observed in PD patients relative to DLB patients. However, no significant difference was found for t-α-syn between these two groups.

In general, Aβ42, p-tau, and t-tau were significant for PD with a moderate effect size compared with DLB.

#### PD versus CBD

Three studies (199 PD patients, 32 CBD patients) evaluated the performance of NFL in differentiating PD and CBD patients. The CSF levels of NFL (SMD = −1.421, 95% CI: −1.928 to −0.914) were reduced in PD patients compared with the CBD patients.

Therefore, NFL can significantly distinguish PD from CBD with a large effect size.

### Assessment of heterogeneity

High levels of heterogeneity were found for NFL (PD versus MSA), t-tau and Aβ42 (PD versus DLB), and NFL and Aβ42 (PD versus PSP). Sensitivity analysis showed that the capacity of all biomarkers in CSF was not affected by specific studies. We failed to detect a significant bias in most studies based on funnel plots and Egger’s test.

### Subgroup and meta-regression analyses for potential moderators

The influence of potential moderators on expression levels of biomarkers, including age, disease stage, and analytical methods, were performed by subgroups and meta-regression analyses (Table S12). The detection assay did not influence the consistency of our results except for the real-time quaking-induced conversion (RT-QuIC) detection of t-α-syn, which was used to detect t-α-syn aggregates instead of free α-syn. Subgroup analysis for the de novo cohort also confirmed the stability of our results. Age (*P* = 0 .001) and MMSE (*P* = 0.004) were significant moderators for t-α-syn and Aβ42, while age (*P* = 0.008) was a significant moderator for NFL in distinguishing between PD and controls.

### Accuracy of biomarkers in PD

**Fig. 4:**
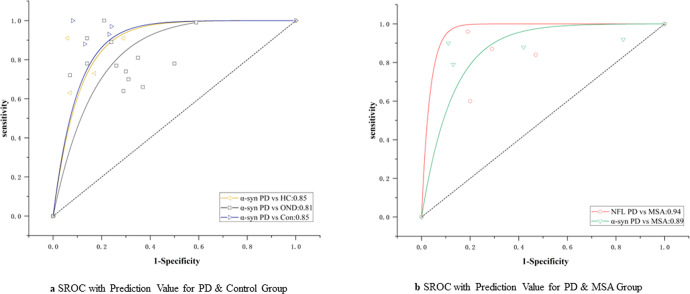
SROC with prediction value for α-syn and NFL in two Groups. **a** SROC with prediction value for PD and control group; **b** SROC with prediction value for PD and MSA. SROC summary receiver operating characteristic curve, PD Parkinson’s disease, MSA multiple system atrophy, α-syn α-synuclein, NFL neurofilament light-chain protein.

For all the biomarkers mentioned above, 18 studies on t-α-syn biomarkers were available to discriminate PD from controls. The pooled sensitivity and specificity were 85% (95% CI = 0.77–0.90) and 74% (95% CI = 0.67–0.80), with an area under the receiver operating characteristic curve (AUC) 0.85 (95% CI = 0.82–0.88) for t-α-syn (Fig. 4). The subgroup analysis of t-α-syn showed that the sensitivity and specificity were 84% (95% CI = 0.66–0.94) and 70% (95% CI = 0.55–0.82) respectively, with AUC 0.81 (95% CI = 0.77–0.84), when other neurological diseases were used as controls. We failed to detect a significant bias in most studies based on funnel plots (*P* > 0.05).

Four studies each on t-α-syn and NFL were available to discriminate PD from MSA. The pooled sensitivity and specificity were 89 % (95% CI = 0.82–0.93) and 66% (95% CI = 0.30–0.90), with AUC 0.89 (95% CI = 0.86–0.92) for t-α-syn. For NFL, the pooled sensitivity and specificity were 98 % (95% CI = 0.89–1.00) and 84% (95% CI = 0.74–0.91), with AUC 0.94 (95% CI = 0.92–0.96) (Fig. 4). Due to the limited number of studies, we failed to conduct a diagnostic meta-analysis on t-α-syn and NFL in PD versus PSP, PD versus DLB, and PD versus CBD groups. Moreover, we failed to conduct a diagnostic analysis for other potential biomarkers in other groups due to the low number of included studies.

However, the HSROC curve of the pooled data showed that the diagnostic effectiveness of t-α-syn was influenced by different cut-off values (*P* < 0.05); while NFL was not influenced by different cut-off values. We failed to detect a significant bias in most studies based on funnel plots (*P* > 0.05).

### Recommendation for CSF biomarkers

According to the diagnostic utility and included number of studies, we found that t-α-syn showed a good performance in distinguishing between PD and controls. NFL was a good biomarker for the differential diagnosis of PD from all APSs except DLB; however, Aβ42 could bridge the gap. Therefore, we believe that the combination of t-α-syn, NFL, and Aβ42 could be promising for the diagnosis and differential diagnosis of PD (Fig. 3).

## Discussion

To the best of our knowledge, this study is the most comprehensive meta-analysis providing an updated summary and evaluation of various CSF biomarkers in PD patients. Our meta-analysis found significant differences in the reported levels of multiple CSF biomarkers between patients with PD and patients with other APSs or controls. Specifically, we demonstrated that DJ-1, t-α-syn, p-α-syn, o-α-syn, Zn, and YKL-40 in CSF could distinguish PD from control samples, while NFL, YKL-40, t-tau, and CRP showed good performances in distinguishing between PD and MSA. Moreover, NFL and Aβ42 could distinguish between PD and PSP. Furthermore, Aβ42, t-tau, and p-tau could differentiate PD from DLB, and NFL could differentiate PD from CBD. Based on the diagnostic utility and the included number of studies, we believe that a combination of t-α-syn, NFL, and Aβ42 could be helpful for the diagnosis and differential diagnosis of PD.

ELISA conditions (e.g., types of antibodies and detection methods) could result in inconsistencies among the published studies. However, the subgroup analysis showed that assay and control types could partially account for the ubiquitous heterogeneity. Our results showed that the Aβ42 levels in CSF were positively correlated with MMSE scores using meta-regression analysis (*P* = 0.008), which was consistent with previous findings demonstrating that Aβ42 was inversely correlated with cognitive decline. Similar results were found with our meta-regression analysis for t-α-syn, which was consistent with previous findings^14^. These findings may indicate that the levels of CSF biomarkers were correlated with disease severity, and these co-occurrence factors could partly account for the underlying heterogeneity.

Our results showed that t-α-syn levels were lower in the PD groups compared with the controls (pooled sensitivity of 85% and a pooled specificity of 74%). Although the absolute values differed among the various immunoassays, our subgroup analysis supported the analytical validity of the immunoassays used to measure t-α-syn in CSF, which increases the possibility of using t-α-syn as a potential biomarker. Furthermore, the RT-QuIC assay used in two included studies has recently emerged as a powerful platform for the amplified detection of disease-associated t-α-syn aggregates^15–17^; however, more studies are needed to confirm their roles in the large sample dataset. For o- and p-α-syn, although the results were meaningful, the number of studies is small; thus, their diagnostic value must be further explored. However, our results failed to find significant differences between PD and APSs (e.g., PSP or DLB), which indicated that CSF t-α-syn values largely overlapped between patients with PD and APS. Therefore, the t-α-syn in CSF might serve as an unspecific marker of synucleinopathy, and its combination with other CSF biomarkers could provide promising diagnostic results.

Our analysis suggested that the NFL in CSF could be beneficial for the differential diagnosis of PD and APSs, although no significant difference in NFL levels was found between PD and control samples, which could be due in part to the less severe and widespread axonal degeneration in PD compared with APSs^11,18^. NFL is a sensitive biomarker of axonal injury; however, it is less specific^19^. Its potential diagnostic value does not depend on the ability to distinguish between neurological disorders featured by a similar degree of axonal loss, but rather, between CNS disorders with a different degree of myelinated axon damage or different rate of disease progression. Considering these, the clinical diagnostic utility of NFL should be complemented with disease-specific biomarkers, as well as neurological assessments.

Core CSF biomarkers of AD pathology (e.g., Aβ42, and t- and p-tau) have also been widely explored in PD. However, our subgroup analysis of PD versus controls showed that p- and t-tau could not distinguish PD from other neurological diseases (e.g., traumatic brain injury, sleep disorder)^20,21^, which may possibly be due to the changes caused by the diseases; thus, p- and t-tau levels may not be the best choice for the diagnosis of PD. Aβ42 in CSF is a validated in vivo marker of Aβ accumulation in AD. Our analysis and several previous studies have demonstrated that reduced Aβ42 is associated with cognitive deterioration and progression of an impaired gait in early PD^22,23^. Although the Aβ42 levels in CSF are significantly associated with PD diagnosis, the AUC was not greater than 0.80^24,25^. Thus, the inclusion of additional biomarkers (e.g., α-syn and NFL) might improve the differential diagnosis utility of CSF biomarkers.

The diagnosis of PD is based on well-defined criteria with excellent sensitivity and specificity in a clinical series; however, the accuracy of the initial diagnosis was only 79.6% even when the assessment was performed by a movement disorder specialist^4^. However, as soon as disease­modifying interventions are available, individuals in pre-symptomatic stages or with a high risk for PD must be immediately identified. Such prodromal PD patients can only be diagnosed with biomarkers (e.g., functional imaging, biofluid and genetic biomarkers) that are currently being evaluated^26–28^. Based on the robust findings of this study, we believe that various combinations of CSF biomarkers can have remarkable diagnostic utilities; however, clinical, and experimental tests are needed to support our conclusions. Large datasets from longitudinal cohorts consisting of clinical and genetic data, neuroimaging, and blood/CSF biomarker analysis (e.g., the Parkinson’s Progression Markers Initiative [PPMI]) could aid in developing predictive PD models^29–31^.

Although we performed a comprehensive literature search and analysis, our meta-analysis had several limitations. First, the literature search was limited to English-language articles. Moreover, some positive biomarkers (e.g., CRP and IL-6) that were identified in individual studies became negative following our comprehensive meta-analysis, which could reflect the lack of quantity, content, and objectives in the individual studies. Furthermore, AD and DLB share many pathological commonalities, the clinical diagnosis can be complicated. Though their identification of them has been considered in our included literature, it is still hard to fully distinguish these two. Thus, more vigorous research is needed to validate our findings. Interestingly, other biomarkers or detection methods may have diagnostic values for PD (e.g., lysosomal enzyme activity, sTREM-2, RT-QuIC assay for α-syn); however, the available data were limited. Therefore, additional studies are needed in the future.

Several biomarkers have been applied in PD for other clinical purposes, including the prediction of disease severity and long-term survival. NFL may identify PD patients with a faster disease progression, enhancing patient selection for clinical trials. However, the information available from the studies included in our analysis was limited, undermining our examination of the underlying mechanisms of potential PD biomarkers and their correlations with other clinical parameters (e.g., disease duration, sleep disturbance, and PD-related genes, such as LRRK2, SNCA, and GBA). Further investigation of the use of combined CSF biomarkers in PD is warranted and may be improved using detailed demographics and clinical variables.

## Methods

Our meta-analysis conformed to the instructions recommended by the Preferred Reporting Items for Systematic Reviews and Meta-Analyses (PRISMA)^32^.

### Search strategy and selection criteria

Two investigators (Xiang and Cong) searched the Web of Science Embase, Cochrane databases, and PubMed (January 1, 1970 to July 1, 2022) for all relevant articles published in English that reported on biomarkers such as amyloid precursor protein metabolism (Aβ42), tau pathology (total tau [t-tau]), synuclein pathology (total α-synuclein [t-α-syn], oligomeric α-synuclein [o-α-syn] and phosphorylated α-synuclein [p-α-syn]), neurodegeneration (neurofilament light chain [NFL], phosphorylated tau [p-tau]), neuroinflammation (C-reactive protein [CRP], IL-6, and YKL-40), and oxidative stress (Cu, Fe, Zn, and Mn) which are present in the CSF of PD patients versus healthy controls (primary outcome) or with APSs (secondary outcome). The lists of all related studies and reviews were examined for broader reports. The search terms were tau or phospho-tau proteins or neurofilament, α-synuclein or β-amyloid42 or amyloid precursor protein or FMS-like tyrosine kinase 3 or ubiquitin-proteasome system or interleukin-6 or neuroinflammation or YKL-40 or axonal degeneration oxidative stress or copper or manganese zinc or iron or c-reactive response protein or DJ-1 or citrulline or arginine AND cerebrospinal fluid AND “Parkinson Disease”[Mesh]” OR Parkinson disease (Supplementary Table 1).

English-language clinical trial (retrospective and prospective) publications, which assessed the roles of all CSF biomarkers, were included in our study only if they met the following criteria: (1) patients were living human beings diagnosed with PD^2,33^; (2) reported data for at least two groups (PD patients and healthy controls); and (3) available literature sources and necessary data (Supplementary Notes2). Quality assessment of the selected studies was performed using the Quality Assessment of Diagnostic Accuracy Studies 2 (QUADAS-2) with a score of 13 as the cut-off for methodological acceptability^34^.

### Data analysis

Data were taken from the baseline of the prospective and cross-sectional studies included. Two investigators independently extracted the mean concentrations ± standard deviations (SD) of the CSF biomarkers and sample sizes from the selected studies as primary data. In addition, the investigators extracted demographic information (age, sex, country, education level, and body mass index [BMI]), clinical features of PD patients (e.g., duration of disease, mean Unified Parkinson’s Disease Rating Scale [UPDRS-III] score, mean Hoehn and Yahr scale, mean Mini-Mental State Examination [MMSE] score) and types of assays (enzyme-linked immunosorbent assay [ELISA] and non-ELISA). Disagreements were resolved through a careful discussion with a third reviewer (Professor Cong Shuyan).

### Statistical analysis

All meta-analyses were performed through STATA 14.0 software (College Station, TX: StataCorp LP, 2013) using the random-effects model. Hedges’ *g*, which was obtained from the standardized mean differences (SMD) based on sample size and mean marker concentrations ± SD, was selected as the final effect size (ES). ES was adjusted for potential biases caused by small sample sizes. Analogous to Cohen’s *d* (Cohen J: Statistical Power Analysis for the Behavioral Sciences, 2013), ES estimates of >0.60, ≥0.30 and <0.60, and ≤0.30 were considered large, moderate, and small, respectively.

The diagnostic accuracy of potential biomarkers was also evaluated by plotting the sensitivity and specificity from individual studies at various cut-off values to create a summary ROC curve. *I*^2^-statistics and *Q* test were performed to evaluate the heterogeneity between studies. The heterogeneity between eligible literature was divided into three categories (0.25, 0.50, and 0.75) that indicated low, moderate, and high heterogeneity levels, respectively^35^. Subgroup analysis was performed to clarify the potential sources of heterogeneity. Meta-regression analyses evaluated ES against potential confounding factors (e.g., clinical characteristics and age). Moreover, a sensitivity test was performed to check the stability of the results. Finally, Egger’s and Funnel tests were employed to check the publication bias of the selected studies. Two-sided *P* < 0.05 was defined as significant.

## Supplementary information

Supplementary Materials

Supplementary Table 4

Dataset 1

## Data Availability

All data analyzed during the current study were acquired from references listed in Supplementary Table 4. Individual participant data that underlie the findings of this study are available upon reasonable request from the corresponding author. The data compromising the privacy of study participants cannot publicly be available.
