# Author Correction: A meta-analysis of the diagnostic utility of biomarkers in cerebrospinal fluid in Parkinson’s disease

**DOI:** 10.1038/s41531-023-00483-3

**Published:** 2023-03-13

**Authors:** Chunchen Xiang, Shengri Cong, Xiaoping Tan, Shuang Ma, Yang Liu, Hailong Wang, Shuyan Cong

**Affiliations:** 1 Department of Neurology, Shengjing Hospital of China Medical University, Shenyang, China; 2 Department of Neurology, Beijing Tiantan Hospital, Capital Medical University, Beijing, China; 3 Department of Clinical Epidemiology and Evidence-Based Medicine, First Hospital of China Medical University, Shenyang, China

**Keywords:** Parkinson's disease, Diagnostic markers

Correction to: *npj Parkinson’s Disease* 10.1038/s41531-022-00431-7, published online 29 November 2022

In this article the paragraph starting ‘Recommendation for CSF biomarkers’ was repeated. The original article has been corrected.

