# Supplementary material for: A meta-analysis of the diagnostic utility of biomarkers in cerebrospinal fluid in Parkinson’s disease
Source: NPJ Parkinsons Dis. 2022 Nov 29;8:165. doi: 10.1038/s41531-022-00431-7 (PMC9709054; doi:10.1038/s41531-022-00431-7)
Supplement: Supplementary file 1 — Supplementary Materials [file 41531_2022_431_MOESM1_ESM.pdf]

**Supplementary Table 1.** Research strategies

| Key-words | Number of full-texts found | | |
|---|---|---|---|
| | Pubmed | Web of Science | Cochrane and Embase |
| ((((parkinson's) OR parkinsons) OR parkinson) OR parkinson's disease) AND cerebrospinal fluid AND α - synuclein | 7013 | 7452 | 2598 |
| ((((parkinson's) OR parkinsons) OR parkinson) OR parkinson's disease) AND cerebrospinal fluid AND amyloid precursor protein | 824 | 835 | 302 |
| ((((parkinson's) OR parkinsons) OR parkinson) OR parkinson's disease) AND cerebrospinal fluid AND β - amyloid42 | 7003 | 8014 | 2789 |
| ((((parkinson's) OR parkinsons) OR parkinson) OR parkinson's disease) AND cerebrospinal fluid AND ubiquitin–proteasome system | 36 | 41 | 20 |
| ((((parkinson's) OR parkinsons) OR parkinson) OR parkinson's disease) AND cerebrospinal fluid AND neuroinflammation | 223 | 238 | 72 |
| ((((parkinson's) OR parkinsons) OR parkinson) OR parkinson's disease) AND cerebrospinal fluid AND YKL-40 | 45 | 59 | 31 |
| ((((parkinson's) OR parkinsons) OR parkinson) OR parkinson's disease) AND cerebrospinal fluid AND Fms-like tyrosine kinase 3 | 8 | 10 | 4 |
| ((((parkinson's) OR parkinsons) OR parkinson) OR parkinson's disease) AND cerebrospinal fluid AND interleukin-6 | 79 | 92 | 39 |
| ((((parkinson's) OR parkinsons) OR parkinson) OR parkinson's disease) AND cerebrospinal fluid AND oxidative stress | 347 | 361 | 132 |
| ((((parkinson's) OR parkinsons) OR parkinson) OR parkinson's disease) AND cerebrospinal fluid AND axonal degeneration | 61 | 79 | 34 |
| ((((parkinson's) OR parkinsons) OR parkinson) OR parkinson's disease) AND cerebrospinal fluid AND tau | 1317 | 1353 | 398 |
| ((((parkinson's) OR parkinsons) OR parkinson) OR parkinson's disease) AND cerebrospinal fluid AND phospho-tau proteins | 79 | 90 | 28 |
| ((((parkinson's) OR parkinsons) OR parkinson) OR parkinson's disease) AND cerebrospinal fluid AND neurofilament | 271 | 284 | 99 |
| ((((parkinson's) OR parkinsons) OR parkinson) OR parkinson's disease) AND cerebrospinal fluid AND copper | 104 | 118 | 48 |
| ((((parkinson's) OR parkinsons) OR parkinson) OR | 62 | 83 | 29 |

| | | | |
|---|---|---|---|
| parkinson’s disease) AND cerebrospinal fluid AND zinc | | | |
| ((((parkinson’s) OR parkinsons) OR parkinson) OR parkinson’s disease) AND cerebrospinal fluid AND iron | 221 | 255 | 93 |
| ((((parkinson’s) OR parkinsons) OR parkinson) OR parkinson’s disease) AND cerebrospinal fluid AND manganese | 89 | 104 | 37 |
| ((((parkinson’s) OR parkinsons) OR parkinson) OR parkinson’s disease) AND cerebrospinal fluid AND C-reactive response protein | 15 | 19 | 8 |
| ((((parkinson’s) OR parkinsons) OR parkinson) OR parkinson’s disease) AND cerebrospinal fluid AND DJ-1 | 95 | 119 | 37 |
| ((((parkinson’s) OR parkinsons) OR parkinson) OR parkinson’s disease) AND cerebrospinal fluid AND Arginine | 39 | 52 | 17 |
| ((((parkinson’s) OR parkinsons) OR parkinson) OR parkinson’s disease) AND cerebrospinal fluid AND Citrulline | 15 | 18 | 9 |
| Total full-texts after removing duplicates | 19326 | 21319 | |

**Supplementary Notes 2.** Extraction form

Listed items:

- Title
- Author
- Year of publication
- Institute
- Country
- Number of patients
- Disease duration
- Inclusion and exclusion criteria
- General characteristics
  - N of patients
  - Age and SD
  - Time since diagnosis and SD (years)
  - % male
  - Mean MMSE and SD
  - Additional information on age across groups
  - Additional information on gender across groups
  - Additional information on disease duration across groups
  - Additional information on UPDRS scores across groups
  - Additional information on HY scores across groups
  - Additional information on MMSE scores across groups
  - Additional information on BMI across groups
  - Additional information on follow-up years across groups
  - Additional information on assay methods across groups

**Supplementary Figure 3**. Quality analysis chart

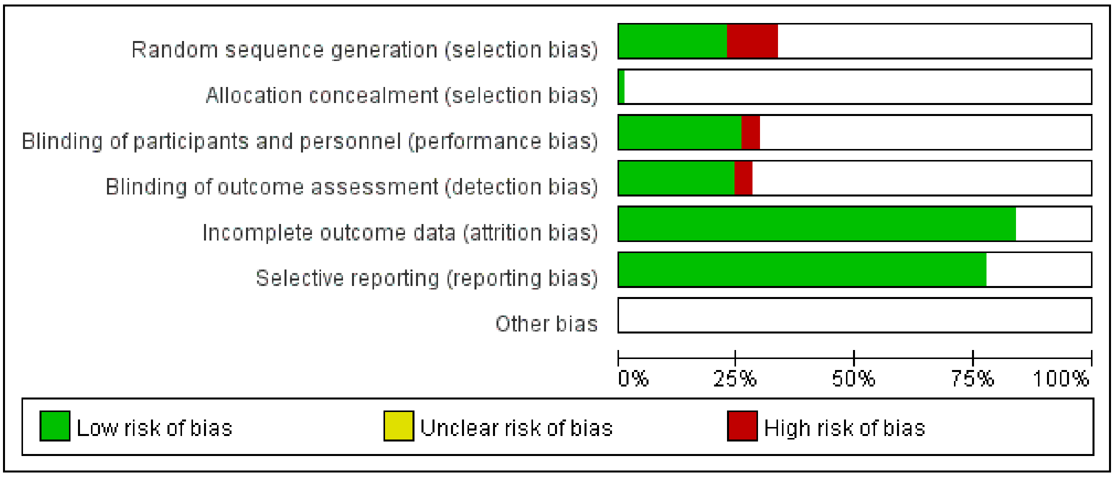

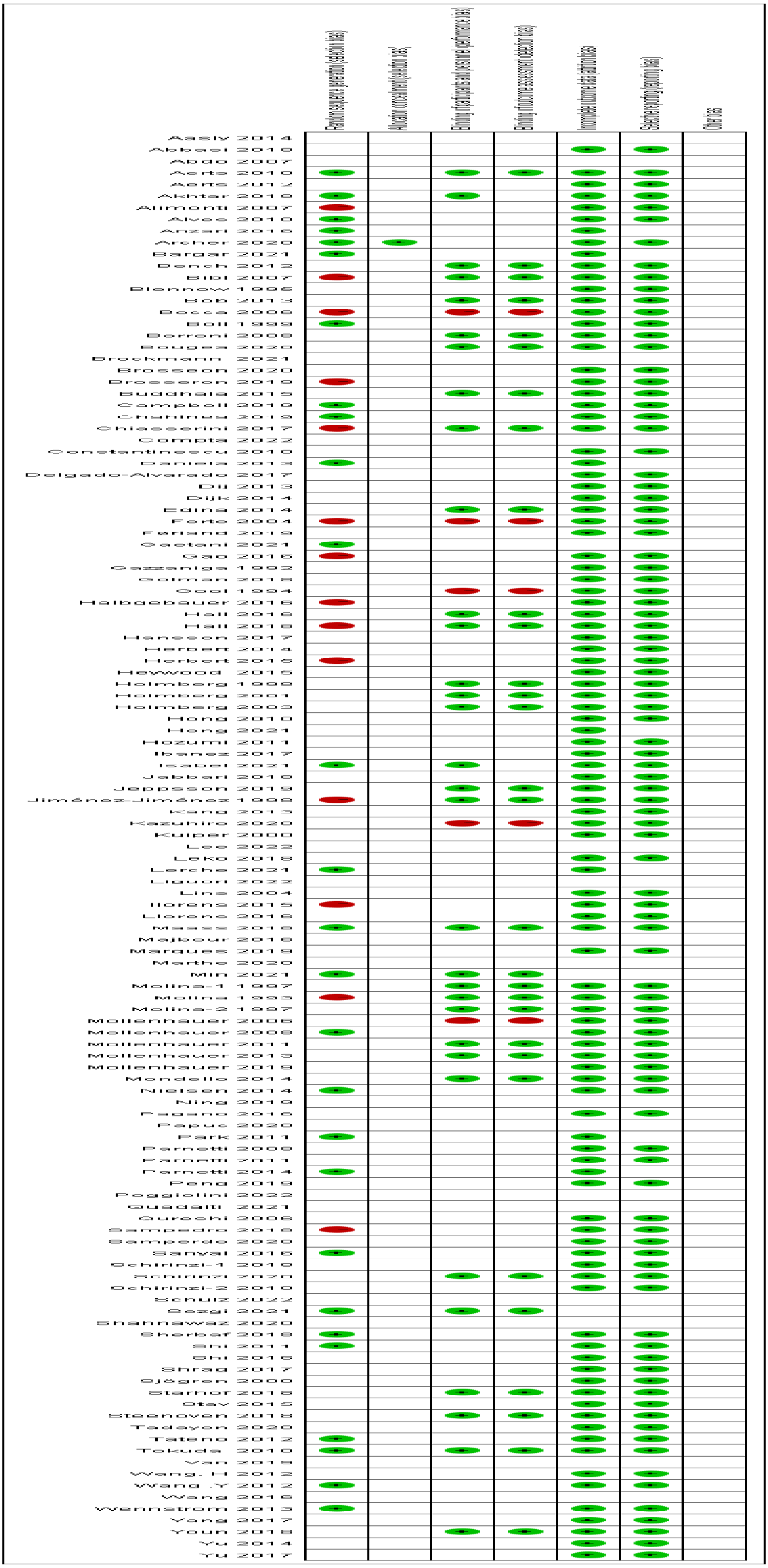

Random sequence generation (selection bias)
Allocation concealment (selection bias)
Blinding of participants and personnel (performance bias)
Blinding of outcome assessment (detection bias)
Incomplete outcome data (attrition bias)
Selective reporting (reporting bias)
Other bias
Aasly 2014
Abbasi 2018
Abdo 2007
Aerts 2010
Aerts 2012
Akhtar 2018
Alimonti 2007
Alves 2010
Anzari 2016
Archer 2020
Bargar 2021
Bench 2012
Bibi 2007
Blennow 1995
Bob 2013
Bocca 2006
Boll 1999
Borroni 2008
Bougea 2020
Brockmann 2021
Brosseon 2020
Brosseron 2019
Buddhala 2015
Campbell 2019
Chahinez 2019
Chiasserini 2017
Compta 2022
Constantinescu 2010
Daniels 2013
Delgado-Alvarado 2017
Dij 2013
Dijk 2014
Edina 2014
Forte 2004
Førland 2019
Gaetani 2021
Gao 2016
Gazzaniga 1992
Golman 2018
Gool 1994
Halbgebauer 2016
Hall 2016
Hall 2018
Hansson 2017
Herbert 2014
Herbert 2015
Haywood 2015
Holmberg 1998
Holmberg 2001
Holmberg 2003
Hong 2010
Hong 2021
Hozumi 2011
Ibanez 2017
Isabel 2021
Jabbari 2018
Jeppsson 2019
Jiménez-Jiménez 1998
Kang 2013
Kazuhiro 2020
Kuiper 2000
Lee 2022
Leko 2018
Lerche 2021
Liguori 2022
Lins 2004
llorens 2015
Llorens 2016
Maass 2018
Majbour 2016
Marques 2019
Marthe 2020
Min 2021
Molina-1 1997
Molina 1993
Molina-2 1997
Mollenhauer 2006
Mollenhauer 2008
Mollenhauer 2011
Mollenhauer 2013
Mollenhauer 2019
Mondello 2014
Nielsen 2014
Ning 2019
Pagano 2010
Papuc 2020
Park 2011
Parnetti 2008
Parnetti 2011
Parnetti 2014
Peng 2019
Poggiolini 2022
Quadalti 2021
Qureshi 2006
Sampedro 2018
Samperdo 2020
Sanyal 2015
Schirinzi-1 2018
Schirinzi 2020
Schirinzi-2 2018
Schulz 2022
Sezgi 2021
Shahnawaz 2020
Sherbaf 2018
Shi 2011
Shi 2015
Shrag 2017
Sjögren 2000
Starhof 2018
Stav 2015
Steenoven 2018
Tadayon 2020
Tateno 2012
Tokuda 2010
Van 2019
Wang. H 2012
Wang .Y 2012
Wang 2016
Wennstrom 2013
Yang 2017
Youn 2018
Yu 2014
Yu 2017

**Supplementary Table 5.** Summary of meta-analytic results

**Table S1 Characteristics of studies included in the meta-analysis for PD vs HC/OND groups.**

| Biomarker | Group | study | n.PD | n.cotrol | Main effect | | | | Heterogeneity | | | | Publication bias |
|---|---|---|---|---|---|---|---|---|---|---|---|---|---|
| | | | | | Measure | ES | 95%CI | P value | I2 | χ2 | df | P value | Egger intercept |
| 1.t-α-syn | 1.PD&HC+OND* | 44 | 7004 | 3380 | SMD | -0.419 | -0.542~-0.295 | 0.000 | 86.2% | 369.65 | 51 | 0.000 | 0.116 |
| | 2.PD&HC* | 36 | 6334 | 2974 | SMD | -0.368 | -0.509~-0.227 | 0.000 | 88.3% | 342.47 | 40 | 0.000 | 0.438 |
| | 3.PD&OND* | 8 | 608 | 364 | SMD | -0.582 | -0.807~-0.356 | 0.000 | 54.2% | 17.48 | 8 | 0.025 | 0.175 |
| 2.p-tau | 1.PD&HC+OND* | 45 | 6636 | 3464 | SMD | -0.297 | -0.373~-0.221 | 0.000 | 60.0% | 124.92 | 50 | 0.000 | 0.188 |
| | 2.PD&HC* | 34 | 5359 | 2795 | SMD | -0.386 | -0.492~-0.281 | 0.000 | 76.2% | 155.36 | 37 | 0.000 | 0.841 |
| | 3.PD&OND | 11 | 572 | 440 | SMD | -0.100 | -0.256~0.056 | 0.207 | 30.2% | 14.32 | 10 | 0.000 | NA |
| 3.t-tau | 1.PD&HC+OND* | 57 | 7368 | 3958 | SMD | -0.274 | -0.349~-0.200 | 0.000 | 64.4% | 171.18 | 61 | 0.000 | 0.032 |
| | 2.PD&HC* | 42 | 6585 | 3267 | SMD | -0.313 | -0.396~-0.231 | 0.000 | 66.2% | 136.18 | 46 | 0.000 | 0.035 |
| | 3.PD&OND | 15 | 892 | 705 | SMD | -0.129 | -0.269~0.012 | 0.072 | 36.9% | 23.77 | 15 | 0.069 | NA |
| 4.Aβ42 | 1.PD&HC+OND* | 52 | 6926 | 3564 | SMD | -0.239 | -0.309~-0.169 | 0.000 | 57.0% | 127.74 | 55 | 0.000 | 0.023 |
| | 2.PD&HC* | 42 | 6238 | 3078 | SMD | -0.262 | -0.341~-0.183 | 0.000 | 61.4% | 116.45 | 45 | 0.000 | 0.009 |
| | 3.PD&OND* | 10 | 758 | 440 | SMD | -0.143 | -0.273~-0.013 | 0.031 | 0.0% | 5.67 | 9 | 0.772 | 0.365 |
| 5.Zn | 1.PD&HC* | 5 | 191 | 172 | SMD | -0.398 | -0.623~-0.173 | 0.001 | 9.6% | 4.42 | 4 | 0.152 | 0.706 |
| 6.DJ-1 | 1.PD&HC* | 2 | 203 | 224 | SMD | -0.791 | -1.380~-0.202 | 0.008 | 88.2% | 8.48 | 1 | 0.004 | NA |
| 7.YKL-40 | 1.PD&HC* | 2 | 257 | 92 | SMD | -0.322 | -0.561~-0.082 | 0.009 | 0.0% | 0.83 | 2 | 0.660 | NA |
| 8.NFL | **1.PD&HC*** | 9 | 927 | 462 | SMD | 0.157 | -0.047~0.360 | 0.131 | 60.9% | 28.16 | 11 | 0.000 | 0.078 |
| 9.o-α-syn | 1.PD&HC+OND* | 8 | 428 | 277 | SMD | 1.616 | 0.888~2.344 | 0.000 | 93.2% | 117.10 | 8 | 0.000 | 0.122 |
| | 2.PD&HC* | 3 | 122 | 145 | SMD | 2.436 | 0.950~3.921 | 0.001 | 94.6% | 55.50 | 3 | 0.000 | 0.908 |
| | 3.PD&OND* | 5 | 306 | 116 | SMD | 0.997 | 0.556~1.439 | 0.000 | 70.5% | 13.56 | 4 | 0.009 | 0.682 |
| 10.p-α-syn | 1. PD&HC+OND* | 2 | 255 | 252 | SMD | 0.439 | 0.259~0.619 | 0.000 | 94.5% | 36.41 | 2 | 0.000 | NA |

**The bolded portion indicates that the biomarker has a higher level in the PD group than in the control group (HC or OND). The * mark indicates positive results. CI confidence interval, Df degrees of freedom.**

**Table S2 Characteristics of studies included in the meta-analysis for PD vs Parkinsonism and AD groups.**

| Group | Biomarker | study | n.PD | n.cotrol | Main effect | | | | Heterogeneity | | | | Publication bias |
|---|---|---|---|---|---|---|---|---|---|---|---|---|---|
| | | | | | Measure | ES | 95%CI | P value | I2 | χ2 | df | P value | Egger intercept |
| 1.PD&MSA | **1.t-a-syn** | 11 | 1114 | 318 | SMD | 0.257 | 0.055~0.459 | 0.013 | 54.4% | 26.33 | 12 | 0.010 | 0.542 |
| | 2.NFL | 7 | 295 | 146 | SMD | -3.069 | -4.545~-1.594 | 0.000 | 95.5% | 133.68 | 6 | 0.000 | 0.011 |
| | 3.t-tau | 5 | 281 | 144 | SMD | -0.977 | -1.520~-0.434 | 0.000 | 82.7% | 23.13 | 4 | 0.000 | 1.000 |
| | **4.Aβ42*** | 2 | 257 | 56 | SMD | 0.284 | -0.006~0.575 | 0.055 | 0.0% | 0.82 | 1 | 0.365 | NA |
| | 5.YKL-40 | 2 | 181 | 61 | SMD | -0.973 | -1.292~-0.655 | 0.000 | 0.0% | 0.28 | 1 | 0.596 | NA |
| | 6.CRP | 2 | 177 | 59 | SMD | -0.556 | -0.871~-0.242 | 0.001 | 0.0% | 0.47 | 1 | 0.493 | NA |
| 2.PD&PSP | **1.Aβ42** | 4 | 521 | 91 | SMD | 0.561 | 0.103~1.018 | 0.016 | 68.4% | 12.66 | 4 | 0.013 | 0.045 |
| | 2.NFL | 6 | 634 | 195 | SMD | -1.509 | -2.222~-0.796 | 0.000 | 92.4% | 91.89 | 7 | 0.000 | 0.097 |
| 3.PD&DLB | **1.Aβ42** | 10 | 839 | 346 | SMD | 0.775 | 0.498~1.052 | 0.000 | 70.6% | 30.58 | 9 | 0.000 | 0.592 |
| | 2.p-tau | 10 | 591 | 283 | SMD | -0.495 | -0.689~-0.301 | 0.000 | 28.0% | 12.50 | 9 | 0.187 | 0.592 |
| | 3.t-tau | 14 | 1023 | 519 | SMD | -0.812 | -1.131~-0.493 | 0.000 | 83.4% | 84.53 | 14 | 0.000 | 0.235 |
| 4.PD&CBD | 1.NFL | 3 | 195 | 24 | SMD | -1.421 | -1.928~-0.914 | 0.000 | 0.0% | 1.65 | 2 | 0.438 | 0.035 |

**The bolded portion indicates that the biomarker has a higher level in the PD group than in the control group ( MSA, PSP, DLB or AD). CI confidence interval, Df degrees of freedom.**

**Supplementary material 6**. Forest plot for Aβ42 in PD& Control group (A), PD&HC group (B), PD&OND group (C), PD&MSA group(D), PD&PSP(E), PD&DLB group(F) displaying effect size (risk ratio) calculated using a random effect model. ES, effective size; CI, confidence intervals; SMD, standardized mean difference.

(A) PD& Control group

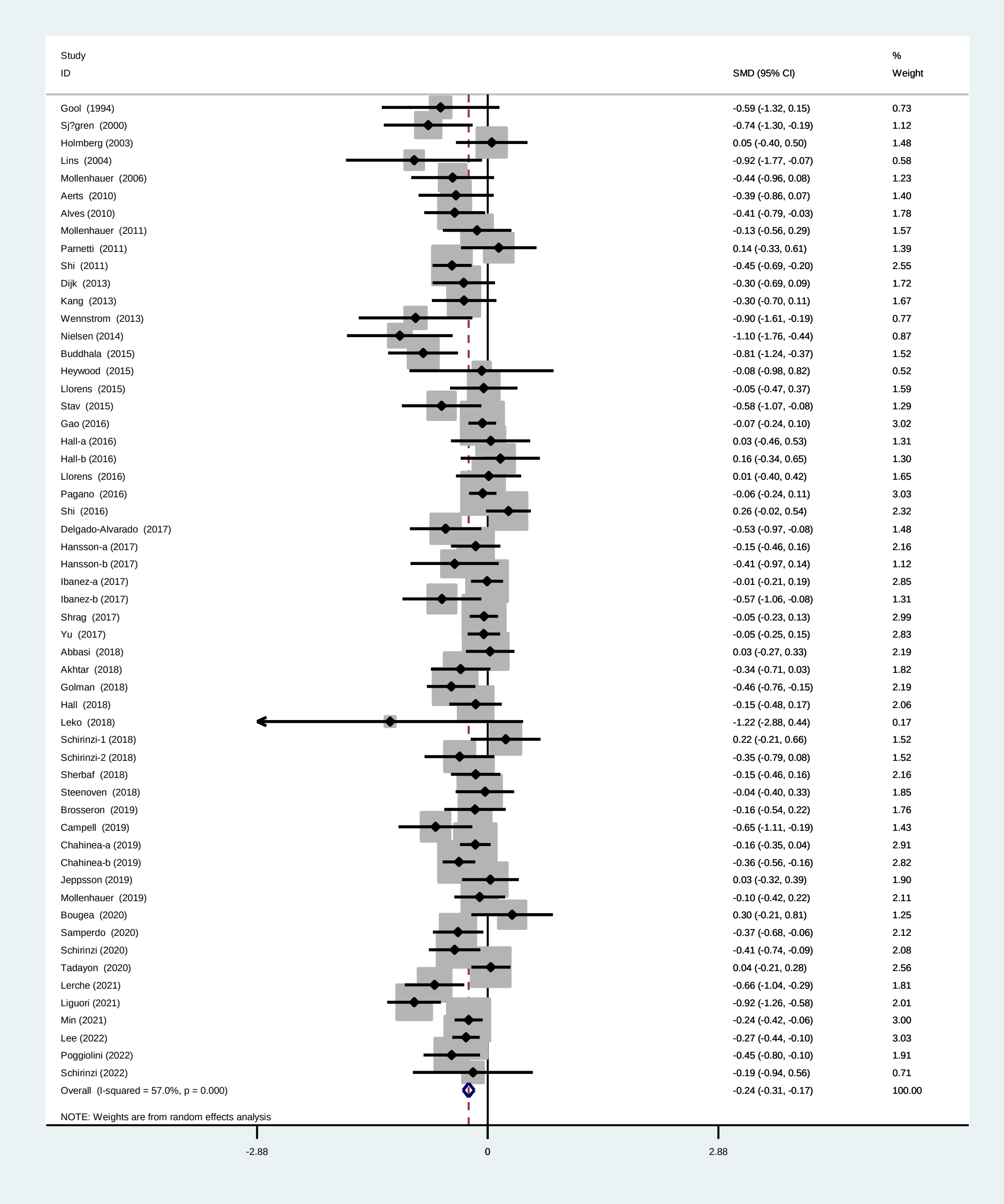

(B) PD&HC group

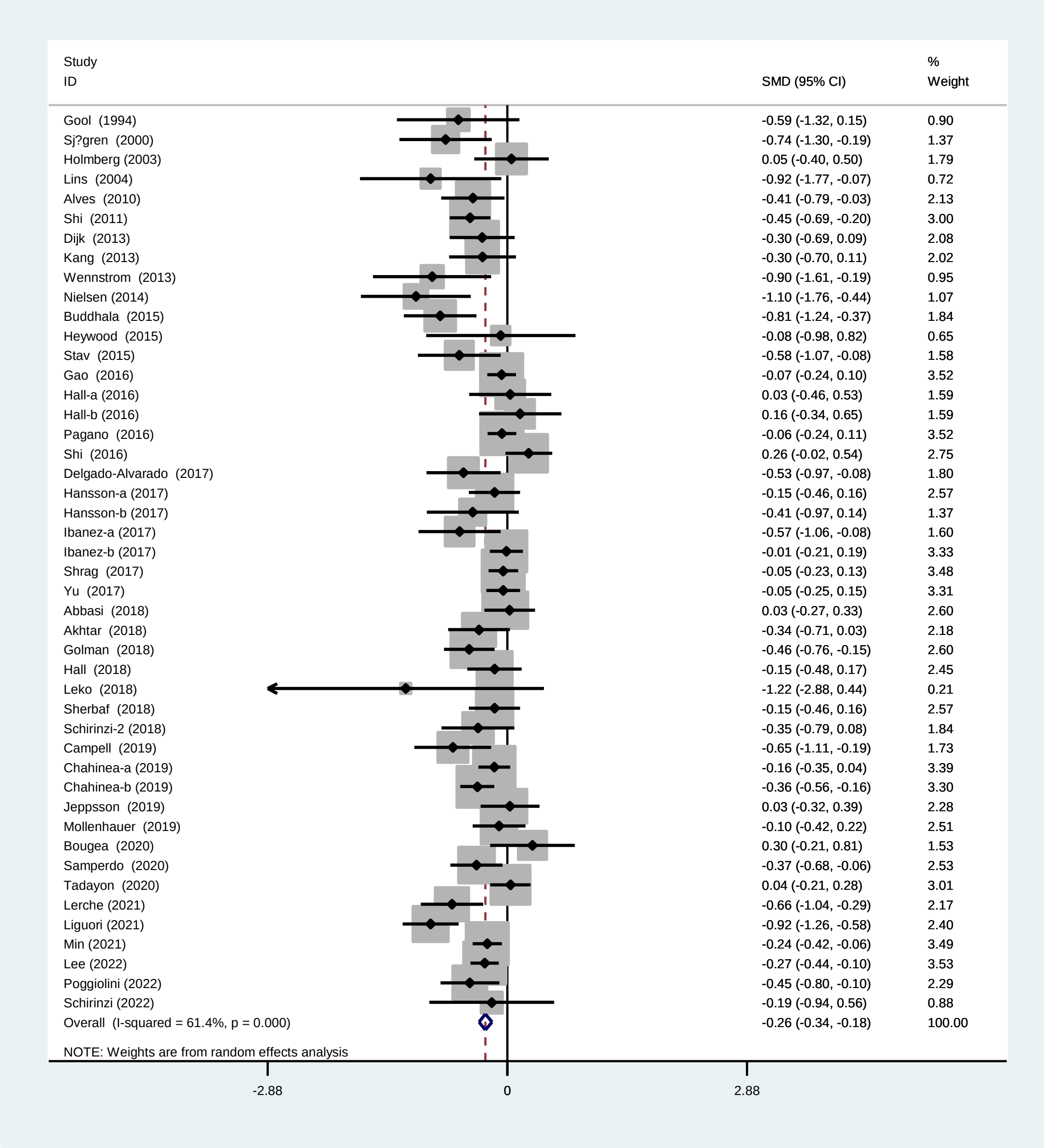
Study
ID
SMD (95% CI)
%
Weight
Gool (1994) -0.59 (-1.32, 0.15) 0.90
Sj?gren (2000) -0.74 (-1.30, -0.19) 1.37
Holmberg (2003) 0.05 (-0.40, 0.50) 1.79
Lins (2004) -0.92 (-1.77, -0.07) 0.72
Alves (2010) -0.41 (-0.79, -0.03) 2.13
Shi (2011) -0.45 (-0.69, -0.20) 3.00
Dijk (2013) -0.30 (-0.69, 0.09) 2.08
Kang (2013) -0.30 (-0.70, 0.11) 2.02
Wennstrom (2013) -0.90 (-1.61, -0.19) 0.95
Nielsen (2014) -1.10 (-1.76, -0.44) 1.07
Buddhala (2015) -0.81 (-1.24, -0.37) 1.84
Heywood (2015) -0.08 (-0.98, 0.82) 0.65
Stav (2015) -0.58 (-1.07, -0.08) 1.58
Gao (2016) -0.07 (-0.24, 0.10) 3.52
Hall-a (2016) 0.03 (-0.46, 0.53) 1.59
Hall-b (2016) 0.16 (-0.34, 0.65) 1.59
Pagano (2016) -0.06 (-0.24, 0.11) 3.52
Shi (2016) 0.26 (-0.02, 0.54) 2.75
Delgado-Alvarado (2017) -0.53 (-0.97, -0.08) 1.80
Hansson-a (2017) -0.15 (-0.46, 0.16) 2.57
Hansson-b (2017) -0.41 (-0.97, 0.14) 1.37
Ibanez-a (2017) -0.57 (-1.06, -0.08) 1.60
Ibanez-b (2017) -0.01 (-0.21, 0.19) 3.33
Shrag (2017) -0.05 (-0.23, 0.13) 3.48
Yu (2017) -0.05 (-0.25, 0.15) 3.31
Abbasi (2018) 0.03 (-0.27, 0.33) 2.60
Akhtar (2018) -0.34 (-0.71, 0.03) 2.18
Golman (2018) -0.46 (-0.76, -0.15) 2.60
Hall (2018) -0.15 (-0.48, 0.17) 2.45
Leko (2018) -1.22 (-2.88, 0.44) 0.21
Sherbaf (2018) -0.15 (-0.46, 0.16) 2.57
Schirinzi-2 (2018) -0.35 (-0.79, 0.08) 1.84
Campell (2019) -0.65 (-1.11, -0.19) 1.73
Chahinea-a (2019) -0.16 (-0.35, 0.04) 3.39
Chahinea-b (2019) -0.36 (-0.56, -0.16) 3.30
Jeppsson (2019) 0.03 (-0.32, 0.39) 2.28
Mollenhauer (2019) -0.10 (-0.42, 0.22) 2.51
Bougea (2020) 0.30 (-0.21, 0.81) 1.53
Samperdo (2020) -0.37 (-0.68, -0.06) 2.53
Tadayon (2020) 0.04 (-0.21, 0.28) 3.01
Lerche (2021) -0.66 (-1.04, -0.29) 2.17
Liguori (2021) -0.92 (-1.26, -0.58) 2.40
Min (2021) -0.24 (-0.42, -0.06) 3.49
Lee (2022) -0.27 (-0.44, -0.10) 3.53
Poggiolini (2022) -0.45 (-0.80, -0.10) 2.29
Schirinzi (2022) -0.19 (-0.94, 0.56) 0.88
Overall (I-squared = 61.4%, p = 0.000) -0.26 (-0.34, -0.18) 100.00
NOTE: Weights are from random effects analysis
-2.88
0
2.88

(C) PD&OND group

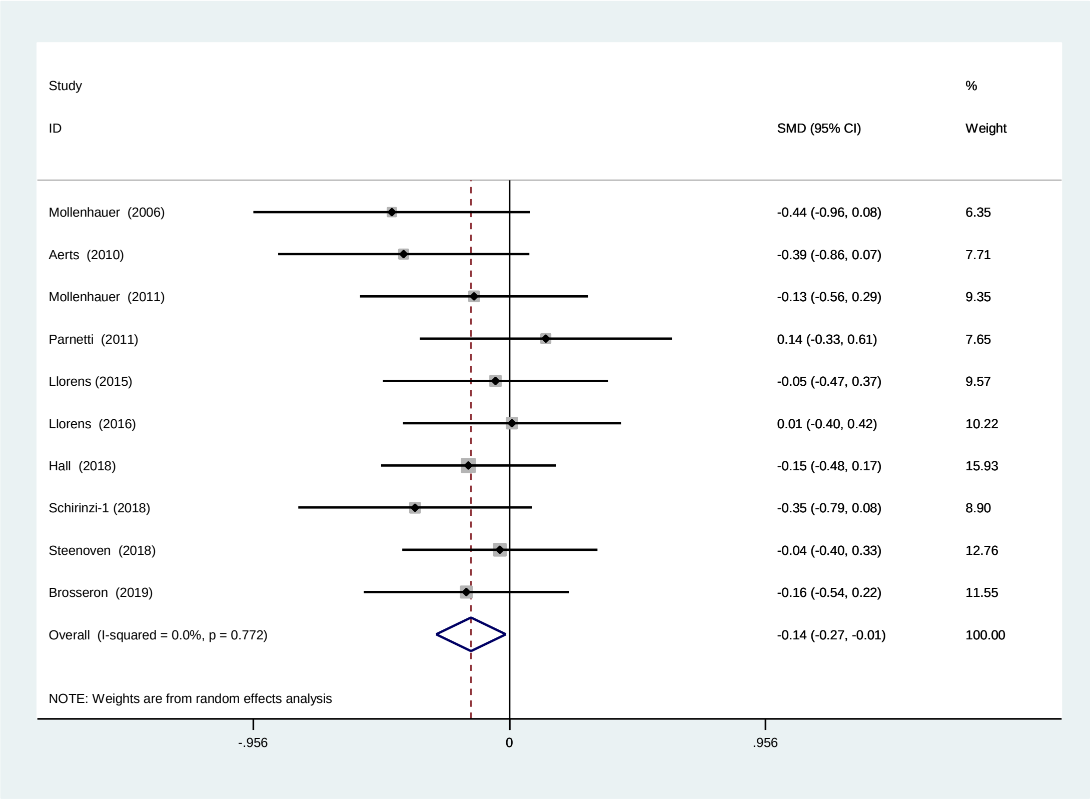


(D) PD&MSA group

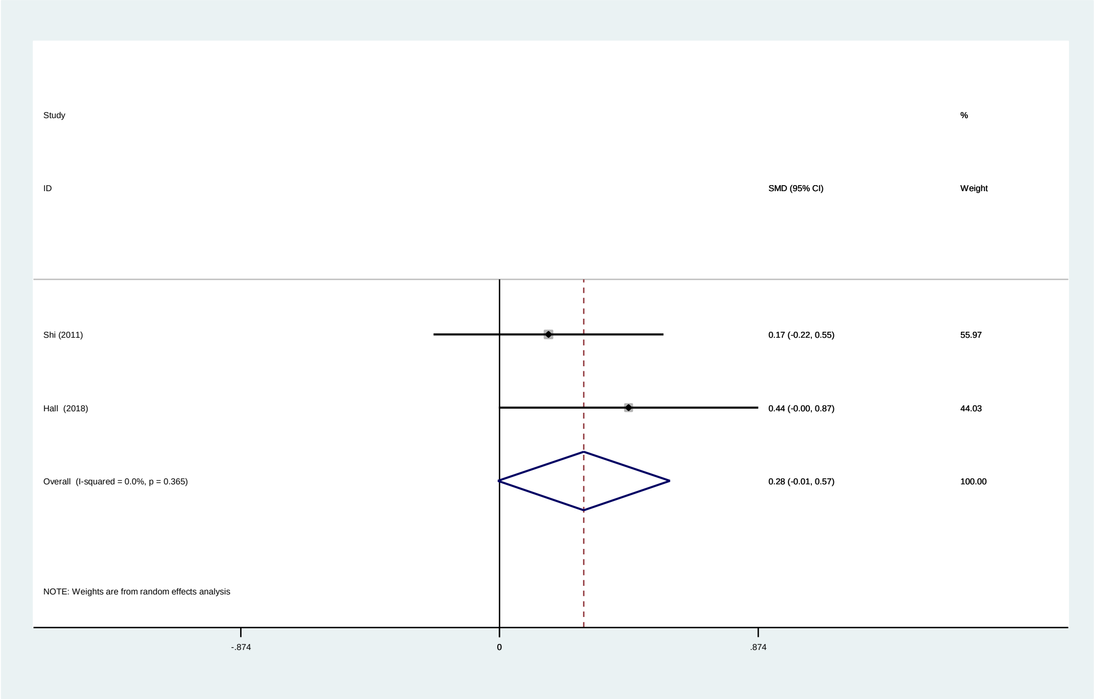

(E) PD&PSP

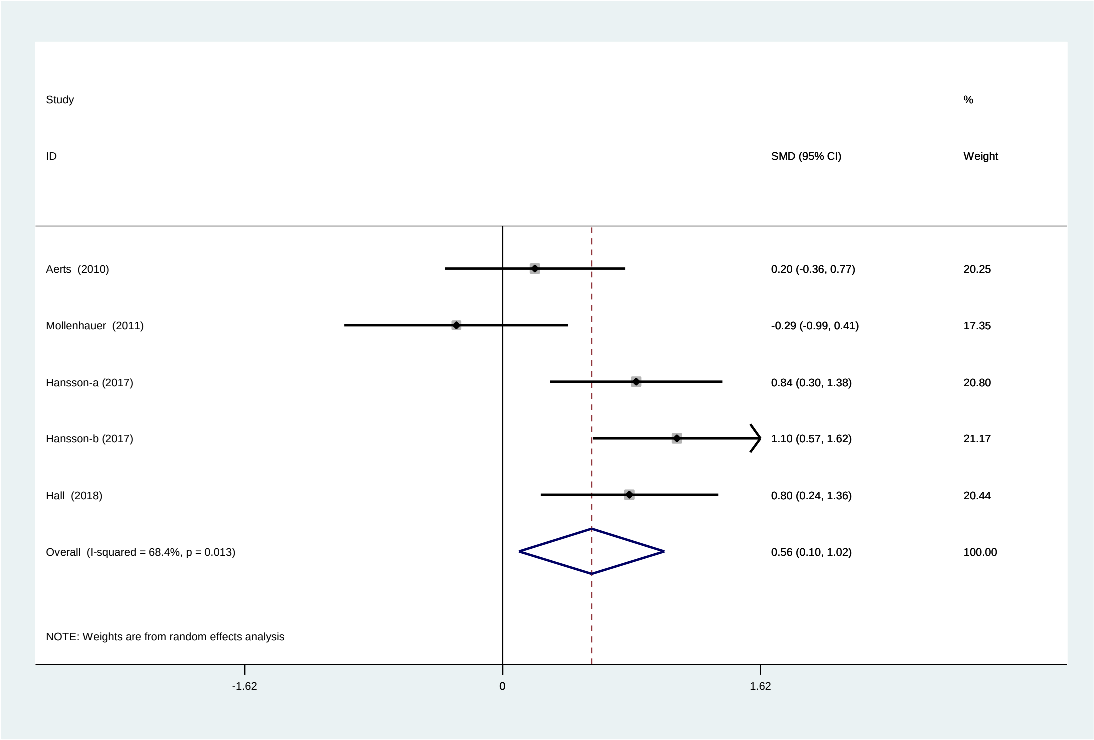


(F) PD&DLB

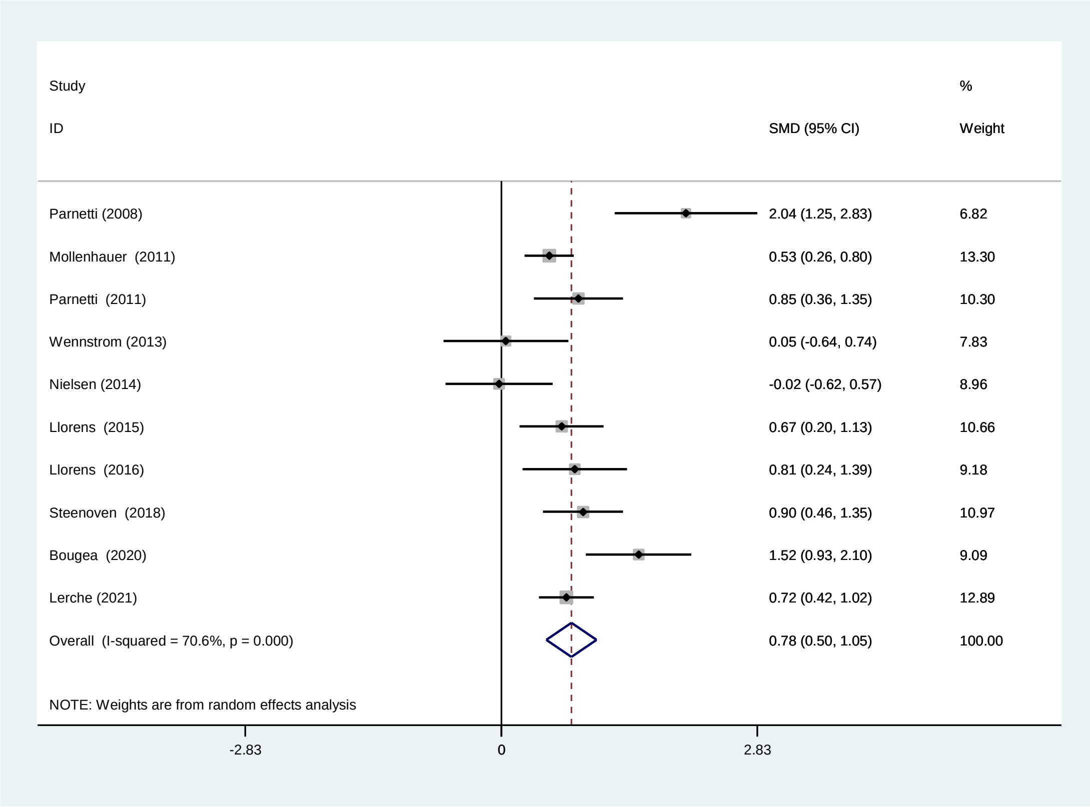

**Supplementary material 7**. Forest plot for t-tau in PD& Control group (A), PD&HC group (B), PD&OND group (C), PD&MSA group(D), PD&PSP(E), PD&DLB group(F) displaying effect size (risk ratio) calculated using a random effect model. ES, effective size; CI, confidence intervals; SMD, standardized mean difference.

(A) PD& Control group

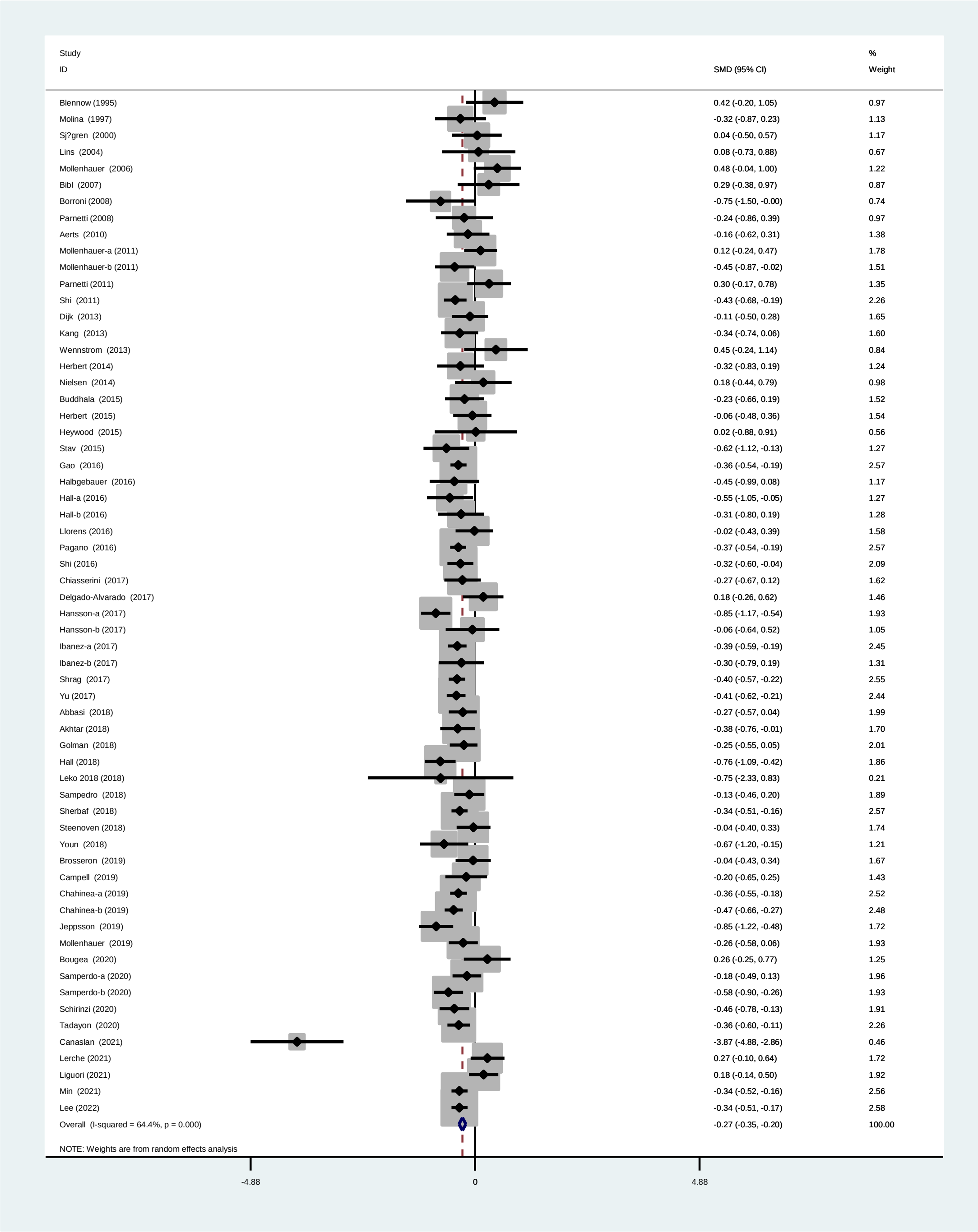

(B) PD&HC group

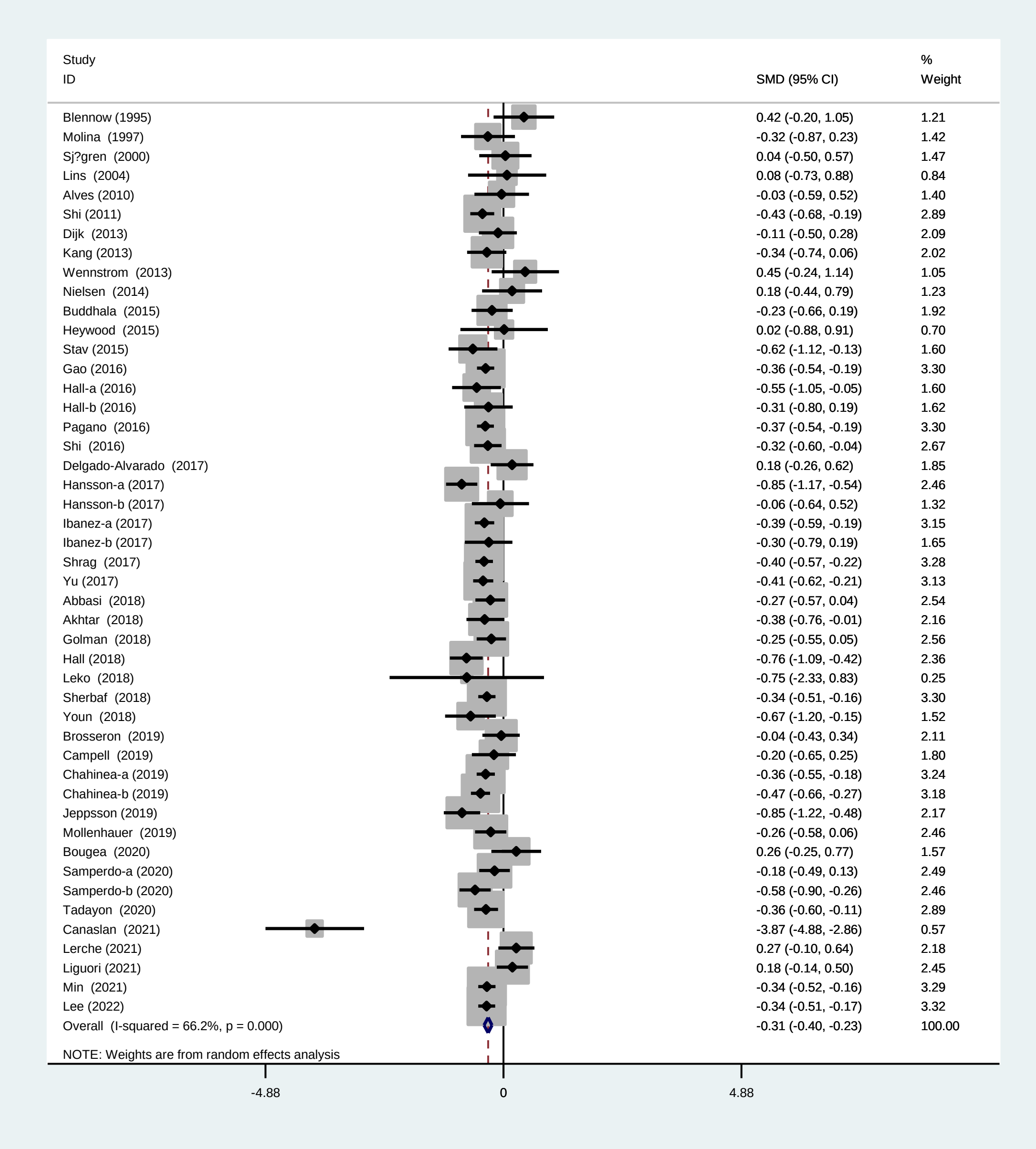
Study
ID
SMD (95% CI)
%
Weight
Blennow (1995) 0.42 (-0.20, 1.05) 1.21
Molina (1997) -0.32 (-0.87, 0.23) 1.42
Sj?gren (2000) 0.04 (-0.50, 0.57) 1.47
Lins (2004) 0.08 (-0.73, 0.88) 0.84
Alves (2010) -0.03 (-0.59, 0.52) 1.40
Shi (2011) -0.43 (-0.68, -0.19) 2.89
Dijk (2013) -0.11 (-0.50, 0.28) 2.09
Kang (2013) -0.34 (-0.74, 0.06) 2.02
Wennstrom (2013) 0.45 (-0.24, 1.14) 1.05
Nielsen (2014) 0.18 (-0.44, 0.79) 1.23
Buddhala (2015) -0.23 (-0.66, 0.19) 1.92
Heywood (2015) 0.02 (-0.88, 0.91) 0.70
Stav (2015) -0.62 (-1.12, -0.13) 1.60
Gao (2016) -0.36 (-0.54, -0.19) 3.30
Hall-a (2016) -0.55 (-1.05, -0.05) 1.60
Hall-b (2016) -0.31 (-0.80, 0.19) 1.62
Pagano (2016) -0.37 (-0.54, -0.19) 3.30
Shi (2016) -0.32 (-0.60, -0.04) 2.67
Delgado-Alvarado (2017) 0.18 (-0.26, 0.62) 1.85
Hansson-a (2017) -0.85 (-1.17, -0.54) 2.46
Hansson-b (2017) -0.06 (-0.64, 0.52) 1.32
Ibanez-a (2017) -0.39 (-0.59, -0.19) 3.15
Ibanez-b (2017) -0.30 (-0.79, 0.19) 1.65
Shrag (2017) -0.40 (-0.57, -0.22) 3.28
Yu (2017) -0.41 (-0.62, -0.21) 3.13
Abbasi (2018) -0.27 (-0.57, 0.04) 2.54
Akhtar (2018) -0.38 (-0.76, -0.01) 2.16
Golman (2018) -0.25 (-0.55, 0.05) 2.56
Hall (2018) -0.76 (-1.09, -0.42) 2.36
Leko (2018) -0.75 (-2.33, 0.83) 0.25
Sherbaf (2018) -0.34 (-0.51, -0.16) 3.30
Youn (2018) -0.67 (-1.20, -0.15) 1.52
Brosseron (2019) -0.04 (-0.43, 0.34) 2.11
Campell (2019) -0.20 (-0.65, 0.25) 1.80
Chahinea-a (2019) -0.36 (-0.55, -0.18) 3.24
Chahinea-b (2019) -0.47 (-0.66, -0.27) 3.18
Jeppsson (2019) -0.85 (-1.22, -0.48) 2.17
Mollenhauer (2019) -0.26 (-0.58, 0.06) 2.46
Bougea (2020) 0.26 (-0.25, 0.77) 1.57
Samperdo-a (2020) -0.18 (-0.49, 0.13) 2.49
Samperdo-b (2020) -0.58 (-0.90, -0.26) 2.46
Tadayon (2020) -0.36 (-0.60, -0.11) 2.89
Canaslan (2021) -3.87 (-4.88, -2.86) 0.57
Lerche (2021) 0.27 (-0.10, 0.64) 2.18
Liguori (2021) 0.18 (-0.14, 0.50) 2.45
Min (2021) -0.34 (-0.52, -0.16) 3.29
Lee (2022) -0.34 (-0.51, -0.17) 3.32
Overall (I-squared = 66.2%, p = 0.000) -0.31 (-0.40, -0.23) 100.00
NOTE: Weights are from random effects analysis
-4.88
0
4.88

(C) PD&OND group

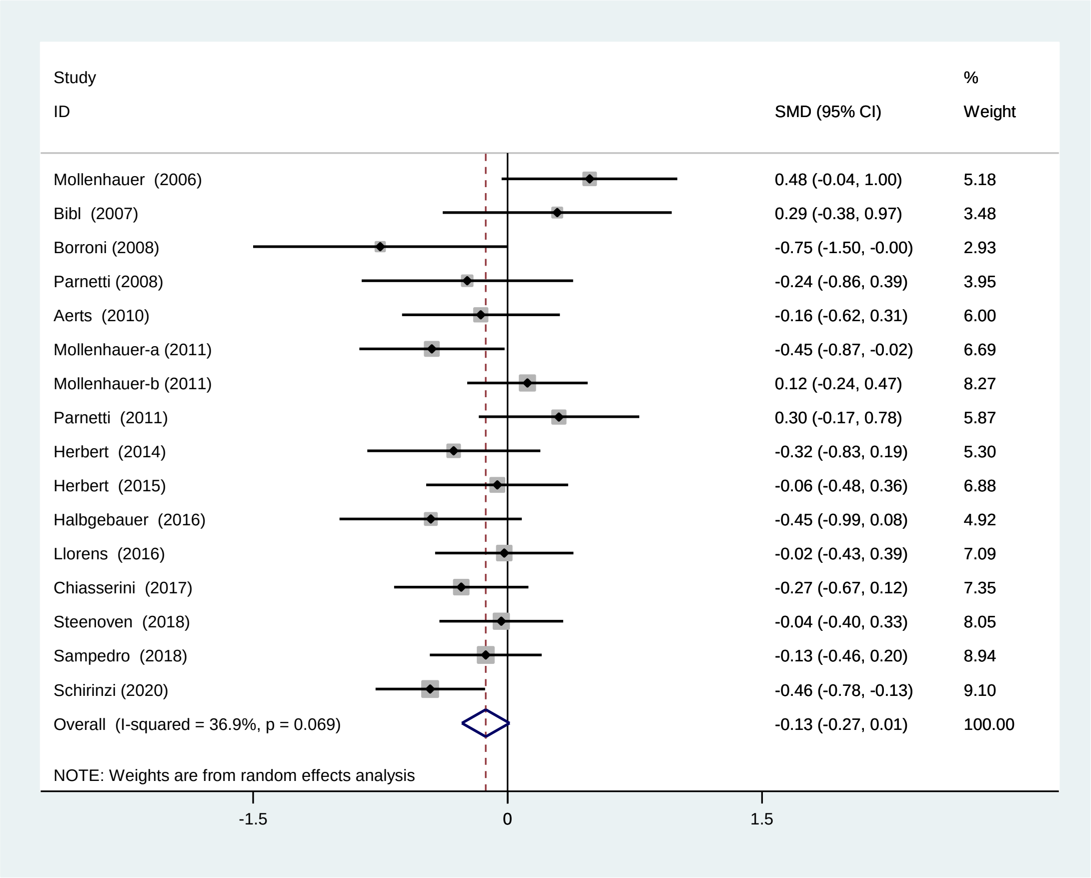


(D) PD&MSA group

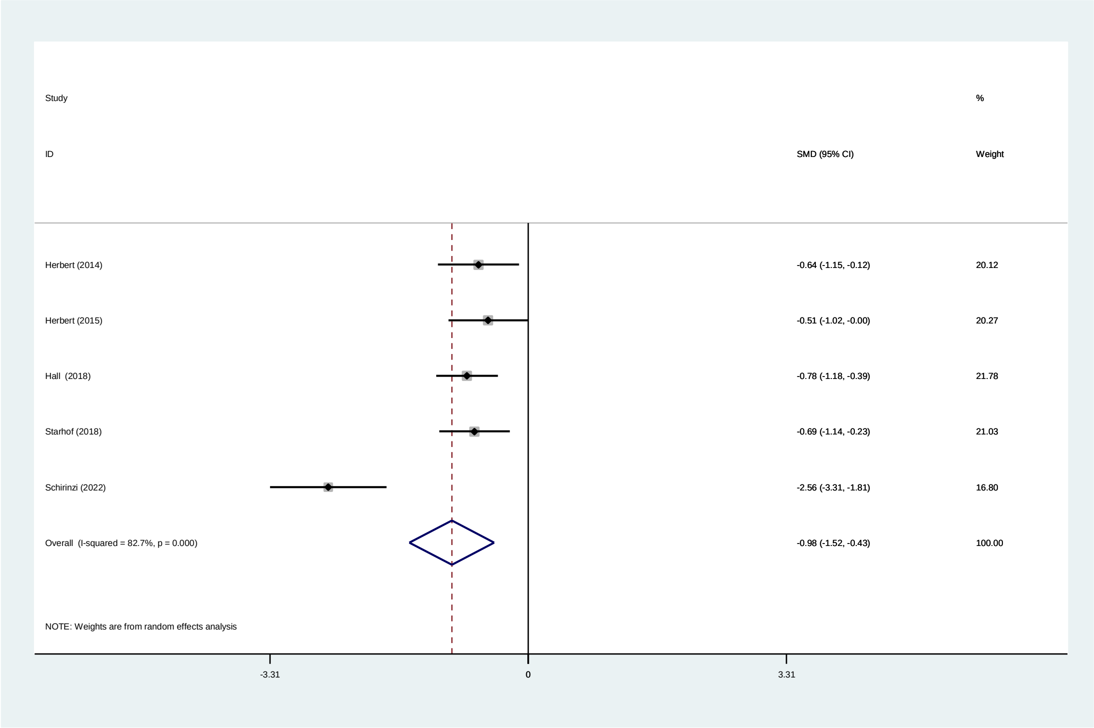

(E) PD&PSP

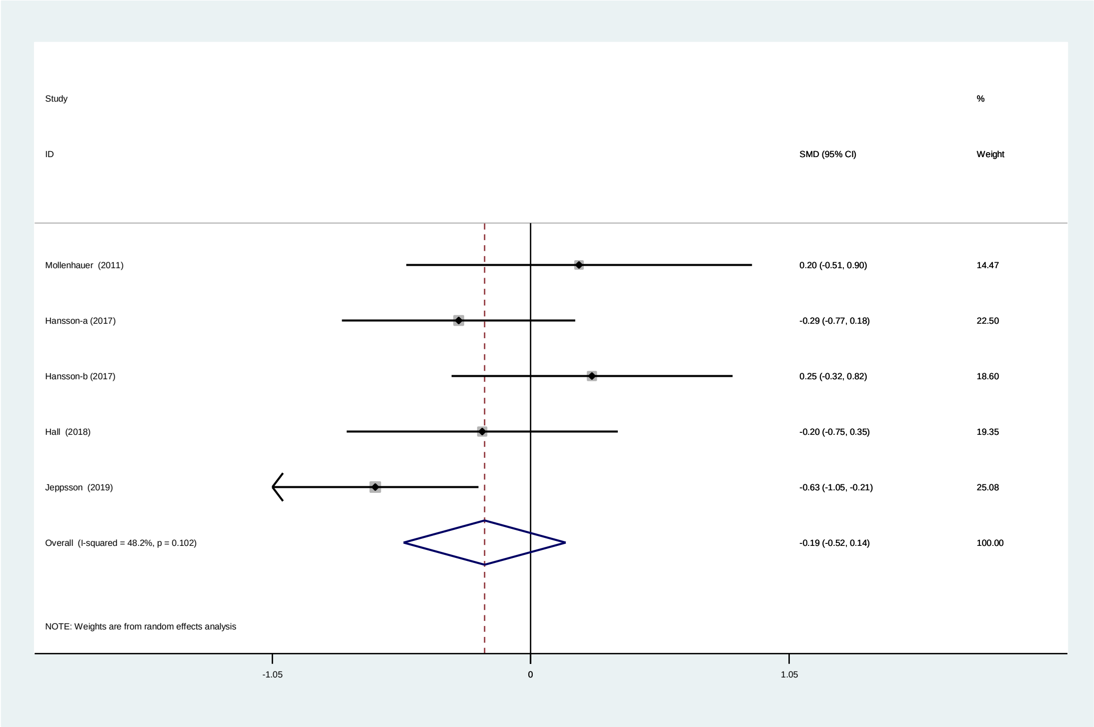


(F) PD&DLB

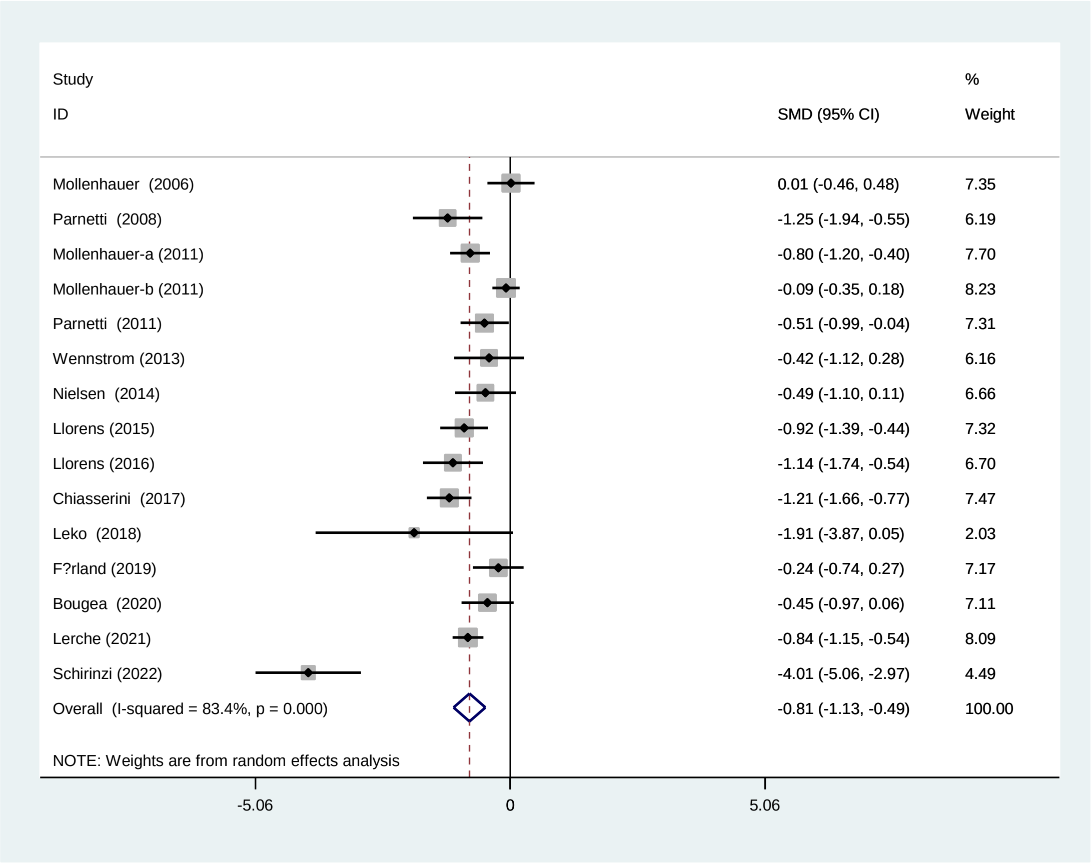

**Supplementary material 8**. Forest plot for p-tau in PD& Control group (A), PD&HC group (B), PD&OND group (C), PD&MSA group(D), PD&PSP(E), PD&DLB group(F) displaying effect size (risk ratio) calculated using a random effect model. ES, effective size; CI, confidence intervals; SMD, standardized mean difference.

(A) PD& Control group

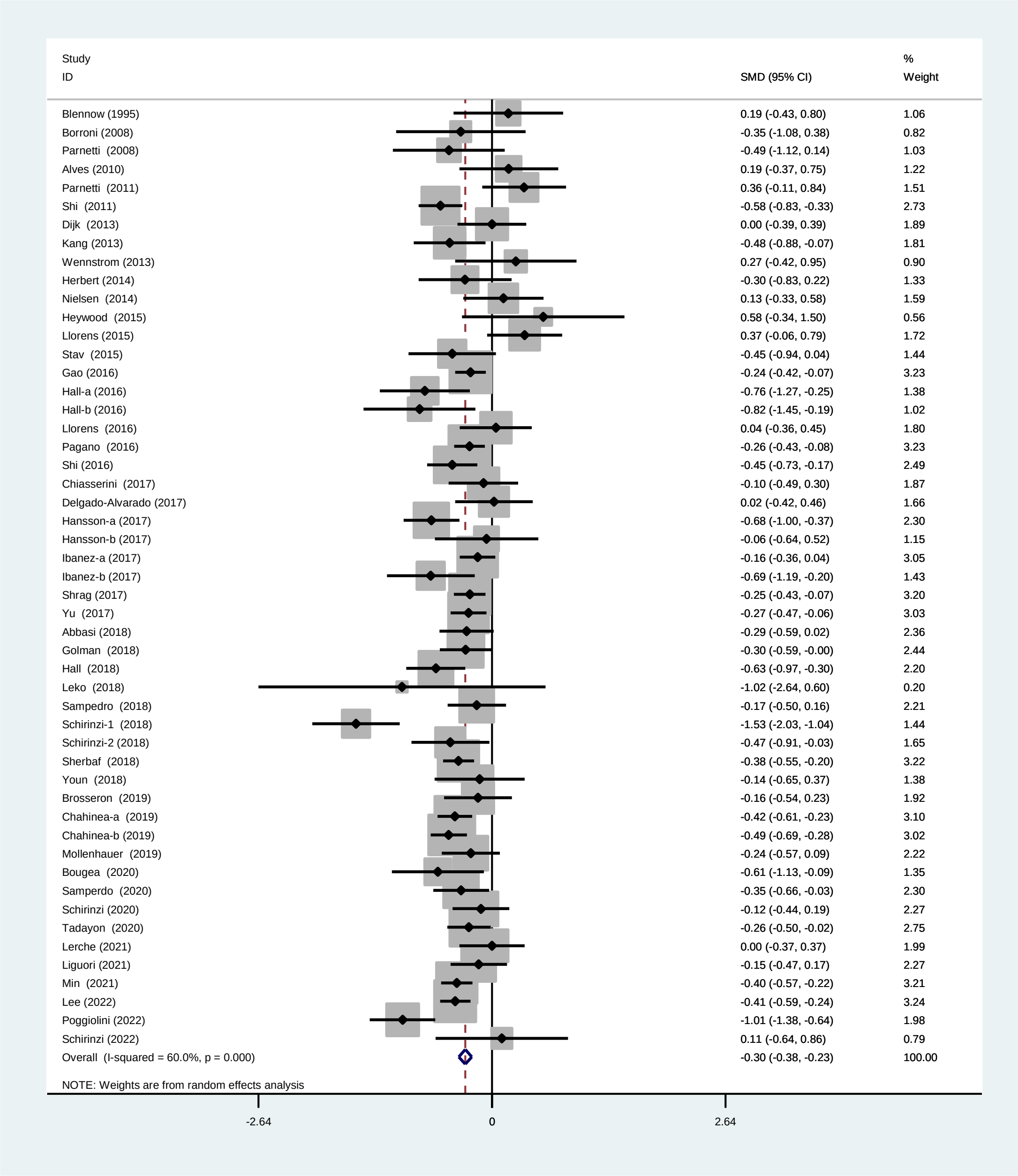

(B) PD&HC group

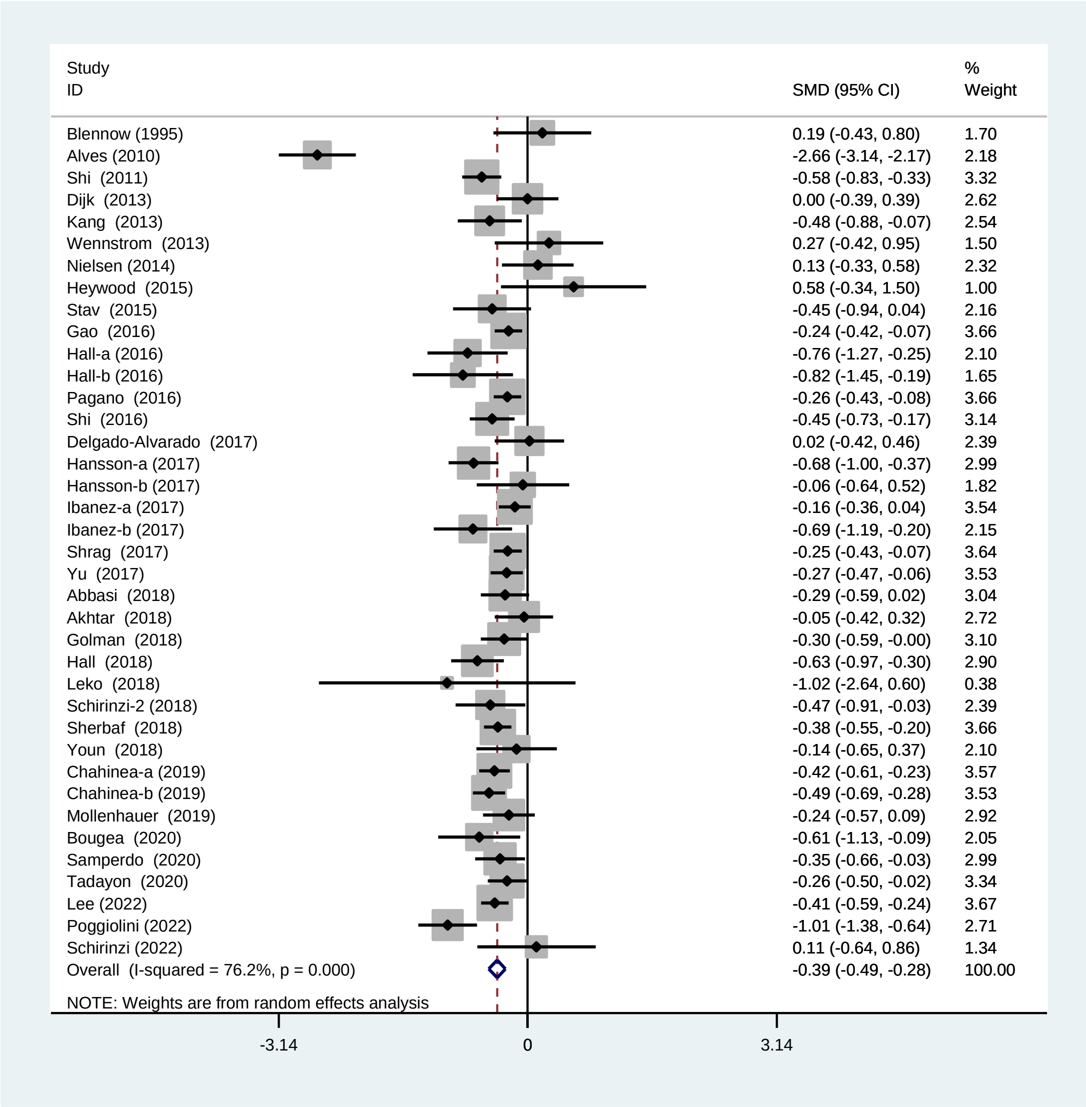
Study
ID
SMD (95% CI)
%
Weight
Blennow (1995) 0.19 (-0.43, 0.80) 1.70
Alves (2010) -2.66 (-3.14, -2.17) 2.18
Shi (2011) -0.58 (-0.83, -0.33) 3.32
Dijk (2013) 0.00 (-0.39, 0.39) 2.62
Kang (2013) -0.48 (-0.88, -0.07) 2.54
Wennstrom (2013) 0.27 (-0.42, 0.95) 1.50
Nielsen (2014) 0.13 (-0.33, 0.58) 2.32
Heywood (2015) 0.58 (-0.34, 1.50) 1.00
Stav (2015) -0.45 (-0.94, 0.04) 2.16
Gao (2016) -0.24 (-0.42, -0.07) 3.66
Hall-a (2016) -0.76 (-1.27, -0.25) 2.10
Hall-b (2016) -0.82 (-1.45, -0.19) 1.65
Pagano (2016) -0.26 (-0.43, -0.08) 3.66
Shi (2016) -0.45 (-0.73, -0.17) 3.14
Delgado-Alvarado (2017) 0.02 (-0.42, 0.46) 2.39
Hansson-a (2017) -0.68 (-1.00, -0.37) 2.99
Hansson-b (2017) -0.06 (-0.64, 0.52) 1.82
Ibanez-a (2017) -0.16 (-0.36, 0.04) 3.54
Ibanez-b (2017) -0.69 (-1.19, -0.20) 2.15
Shrag (2017) -0.25 (-0.43, -0.07) 3.64
Yu (2017) -0.27 (-0.47, -0.06) 3.53
Abbasi (2018) -0.29 (-0.59, 0.02) 3.04
Akhtar (2018) -0.05 (-0.42, 0.32) 2.72
Golman (2018) -0.30 (-0.59, -0.00) 3.10
Hall (2018) -0.63 (-0.97, -0.30) 2.90
Leko (2018) -1.02 (-2.64, 0.60) 0.38
Schirinzi-2 (2018) -0.47 (-0.91, -0.03) 2.39
Sherbaf (2018) -0.38 (-0.55, -0.20) 3.66
Youn (2018) -0.14 (-0.65, 0.37) 2.10
Chahinea-a (2019) -0.42 (-0.61, -0.23) 3.57
Chahinea-b (2019) -0.49 (-0.69, -0.28) 3.53
Mollenhauer (2019) -0.24 (-0.57, 0.09) 2.92
Bougea (2020) -0.61 (-1.13, -0.09) 2.05
Samperdo (2020) -0.35 (-0.66, -0.03) 2.99
Tadayon (2020) -0.26 (-0.50, -0.02) 3.34
Lee (2022) -0.41 (-0.59, -0.24) 3.67
Poggiolini (2022) -1.01 (-1.38, -0.64) 2.71
Schirinzi (2022) 0.11 (-0.64, 0.86) 1.34
Overall (I-squared = 76.2%, p = 0.000) -0.39 (-0.49, -0.28) 100.00
NOTE: Weights are from random effects analysis
-3.14
0
3.14

(C) PD&OND group

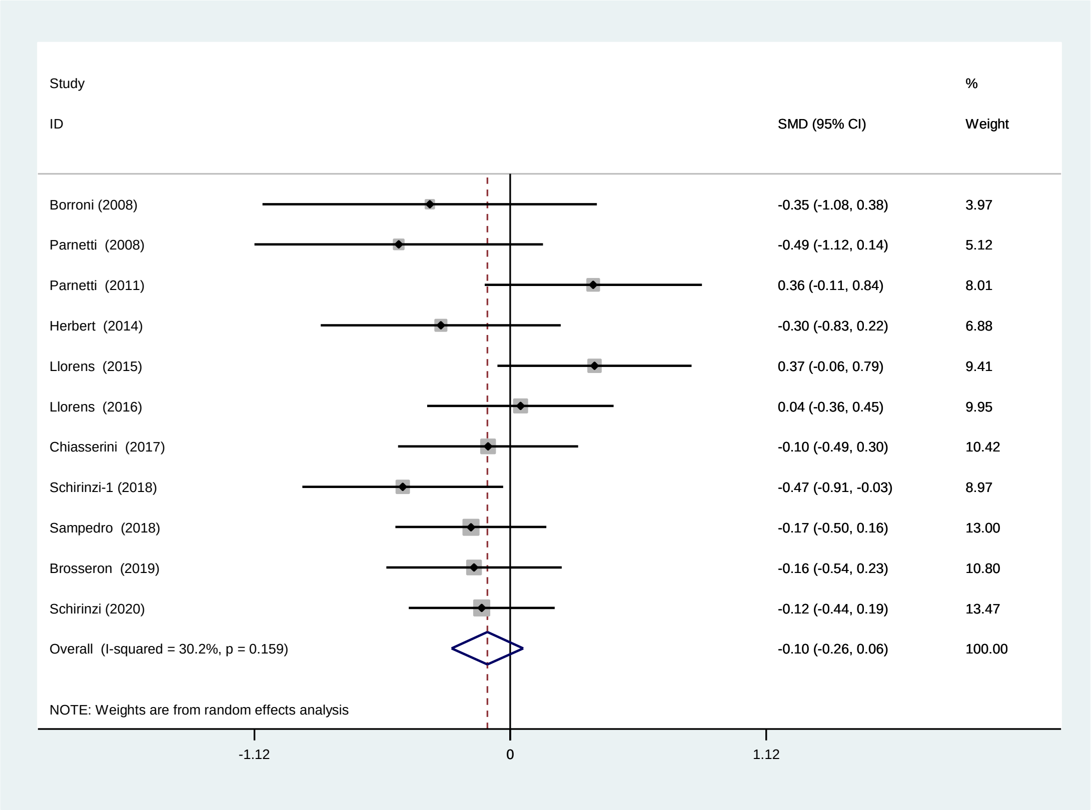


(D) PD&MSA group

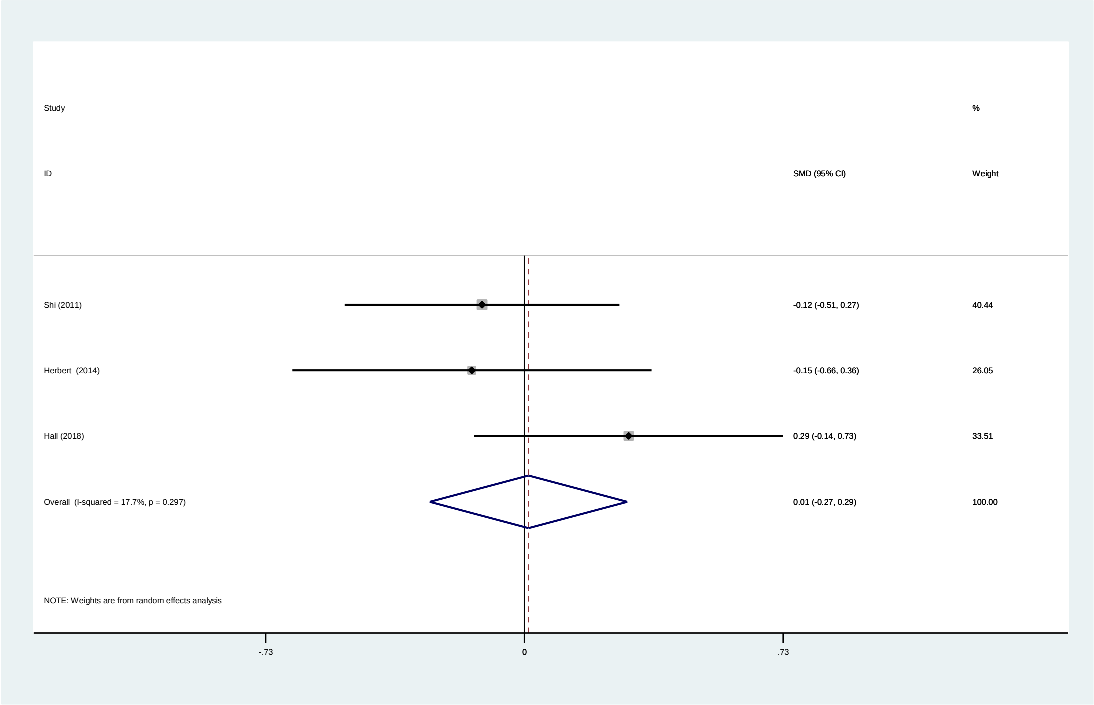

(E) PD&PSP

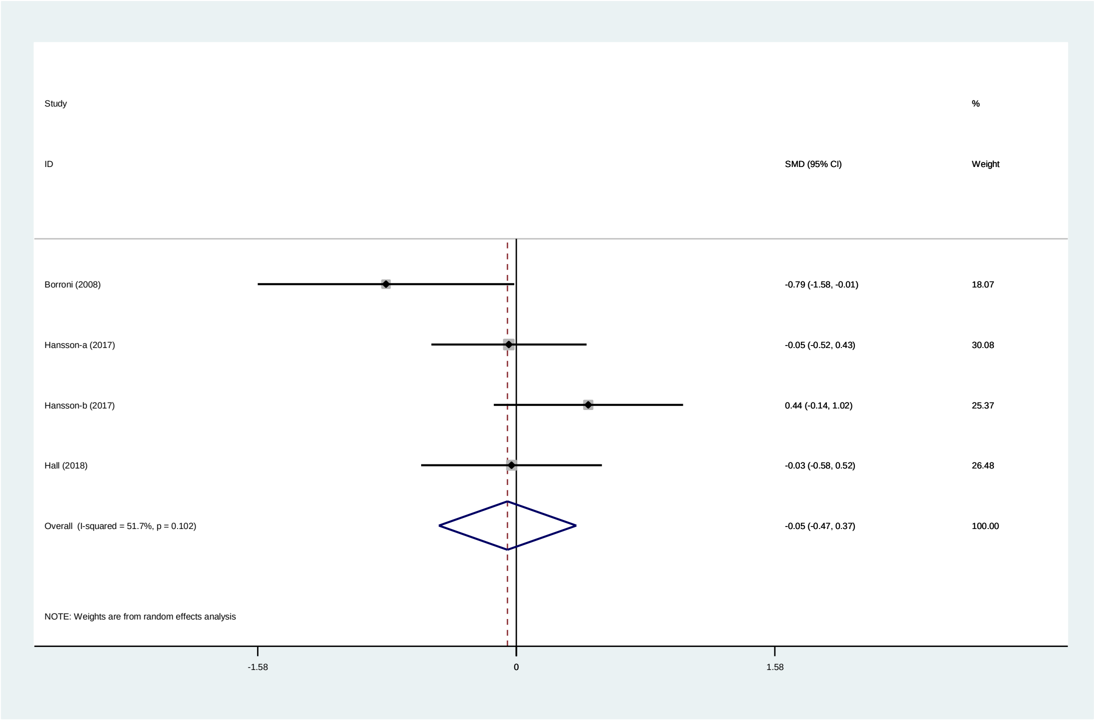


(F) PD&DLB

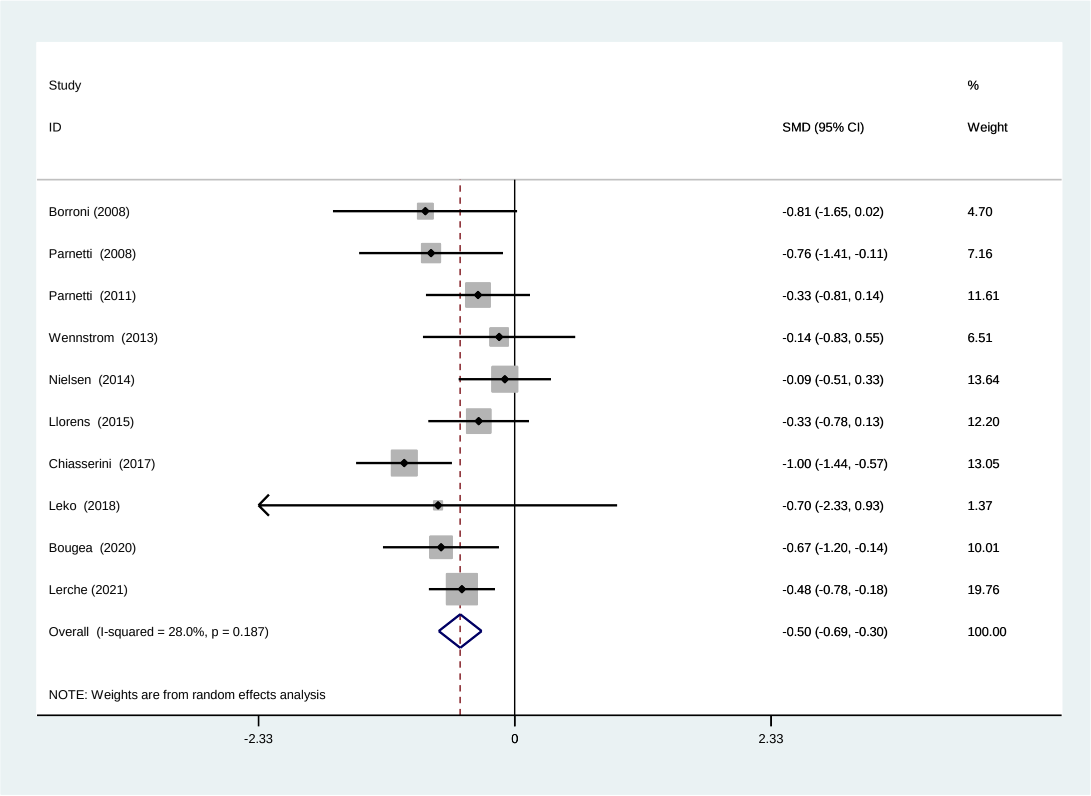

**Supplementary material 9**. Forest plot for NFL in PD&HC group (A), PD&MSA group(B), PD&PSP(C), PD&CBD group(D) displaying effect size (risk ratio) calculated using a random effect model. ES, effective size; CI, confidence intervals; SMD, standardized mean difference.

(A) PD& HC group

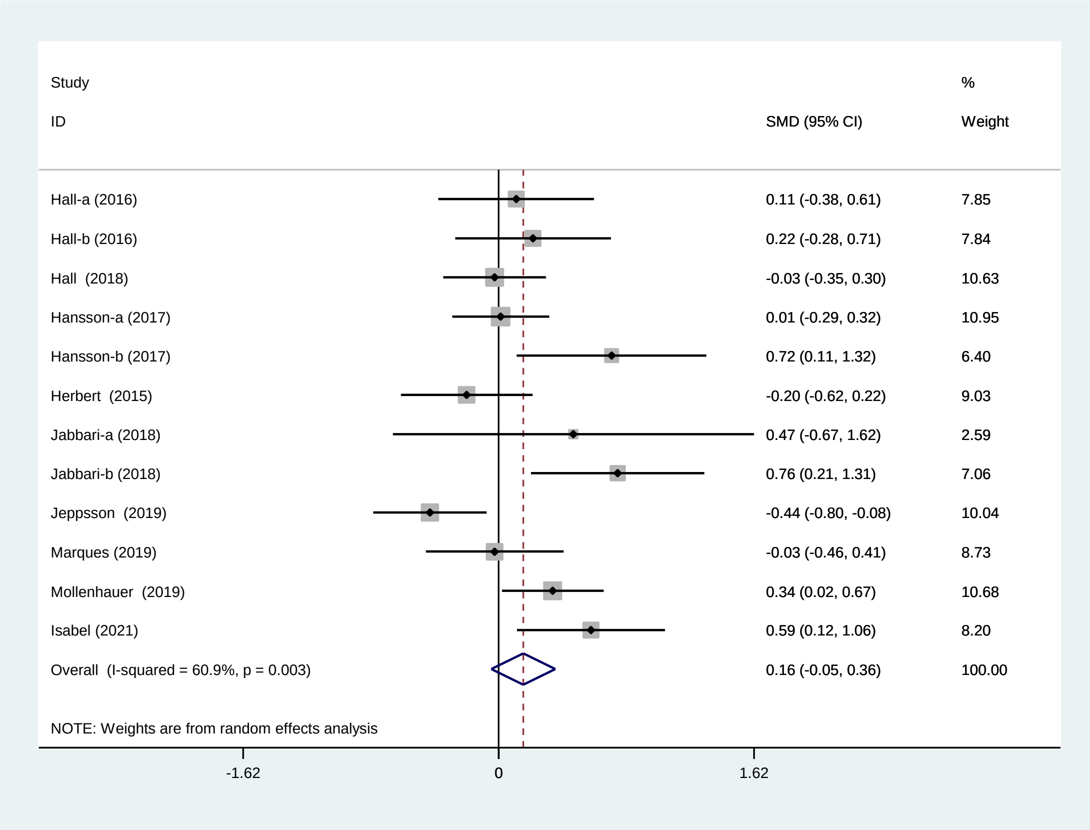


(B) PD&MSA group

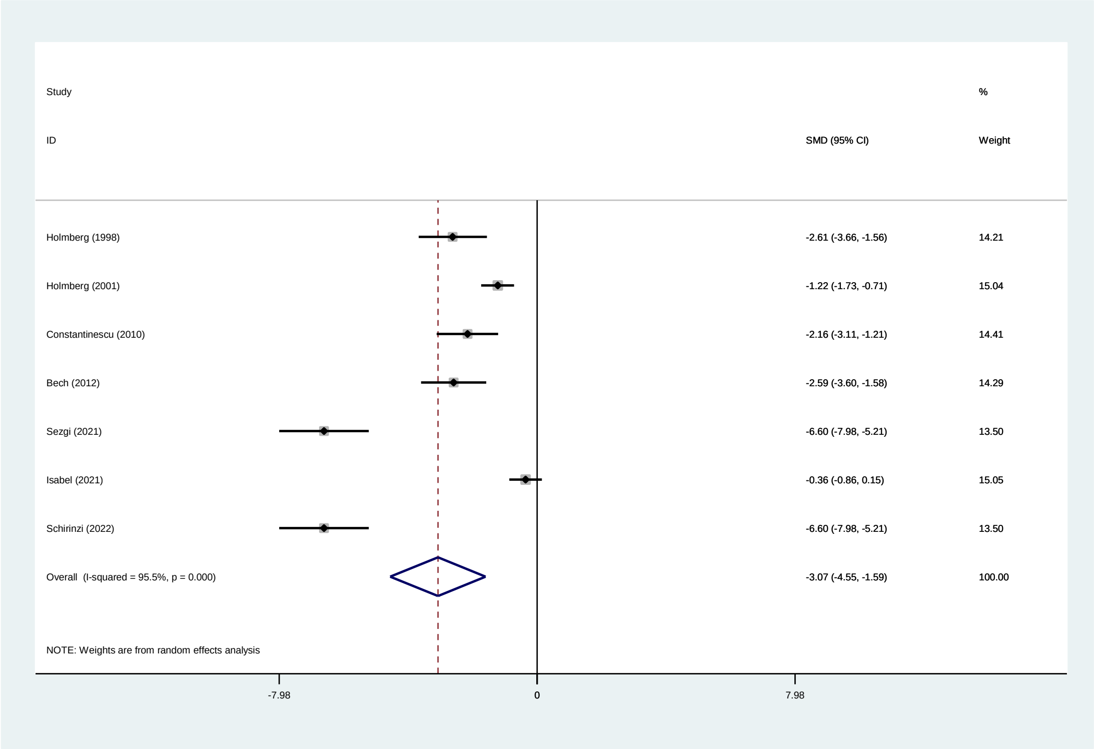

(C) PD&PSP group

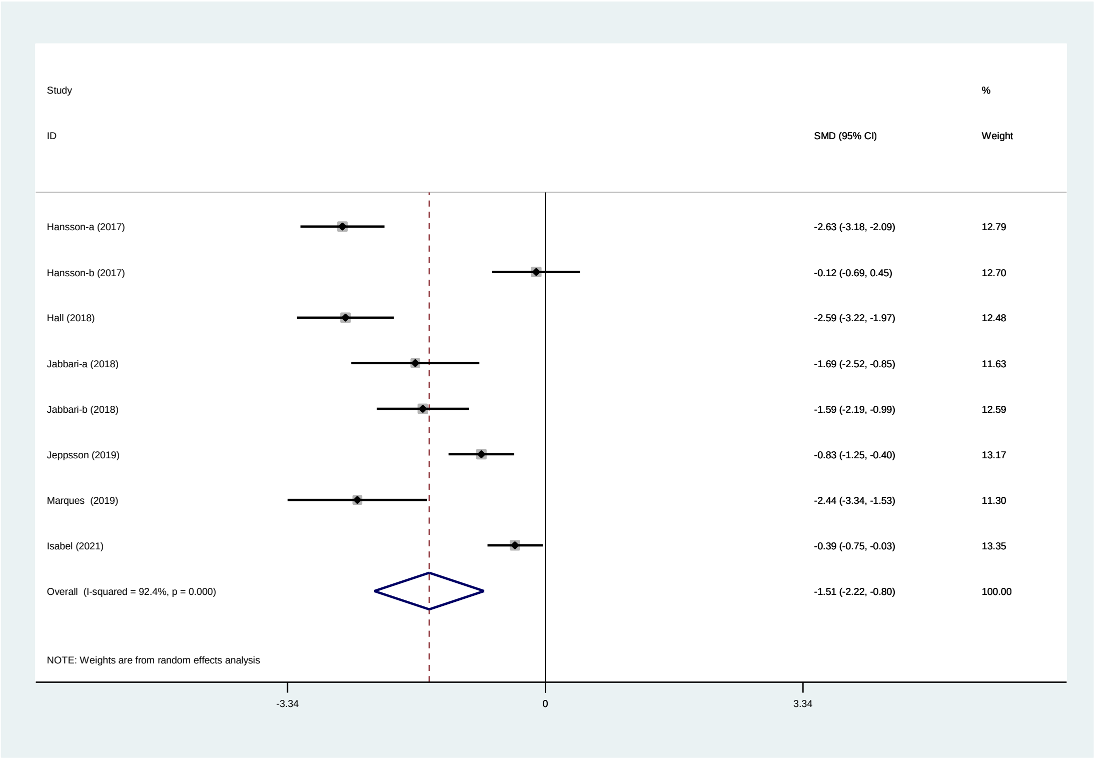


(D) PD&CBD group

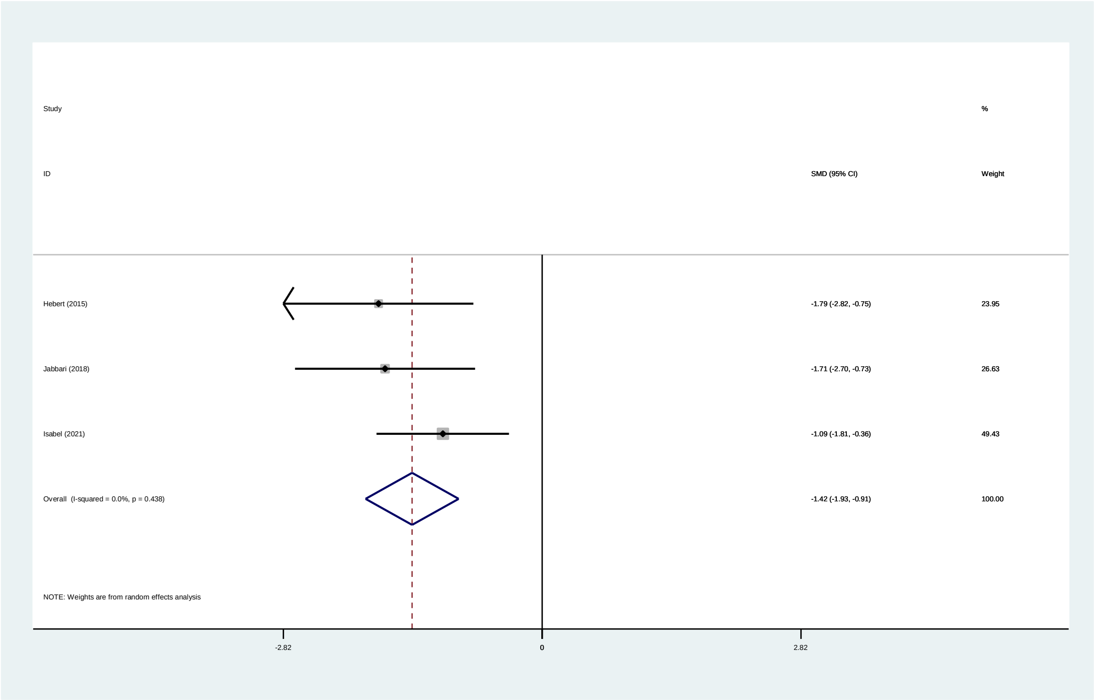

**Supplementary material 10**. Forest plot for t-α-syn in PD& Control group (A), PD&HC group (B), PD&OND group (C), PD&MSA group(D), PD&PSP(E), PD&DLB group(F) displaying effect size (risk ratio) calculated using a random effect model. ES, effective size; CI, confidence intervals; SMD, standardized mean difference.

(A) PD& Control group

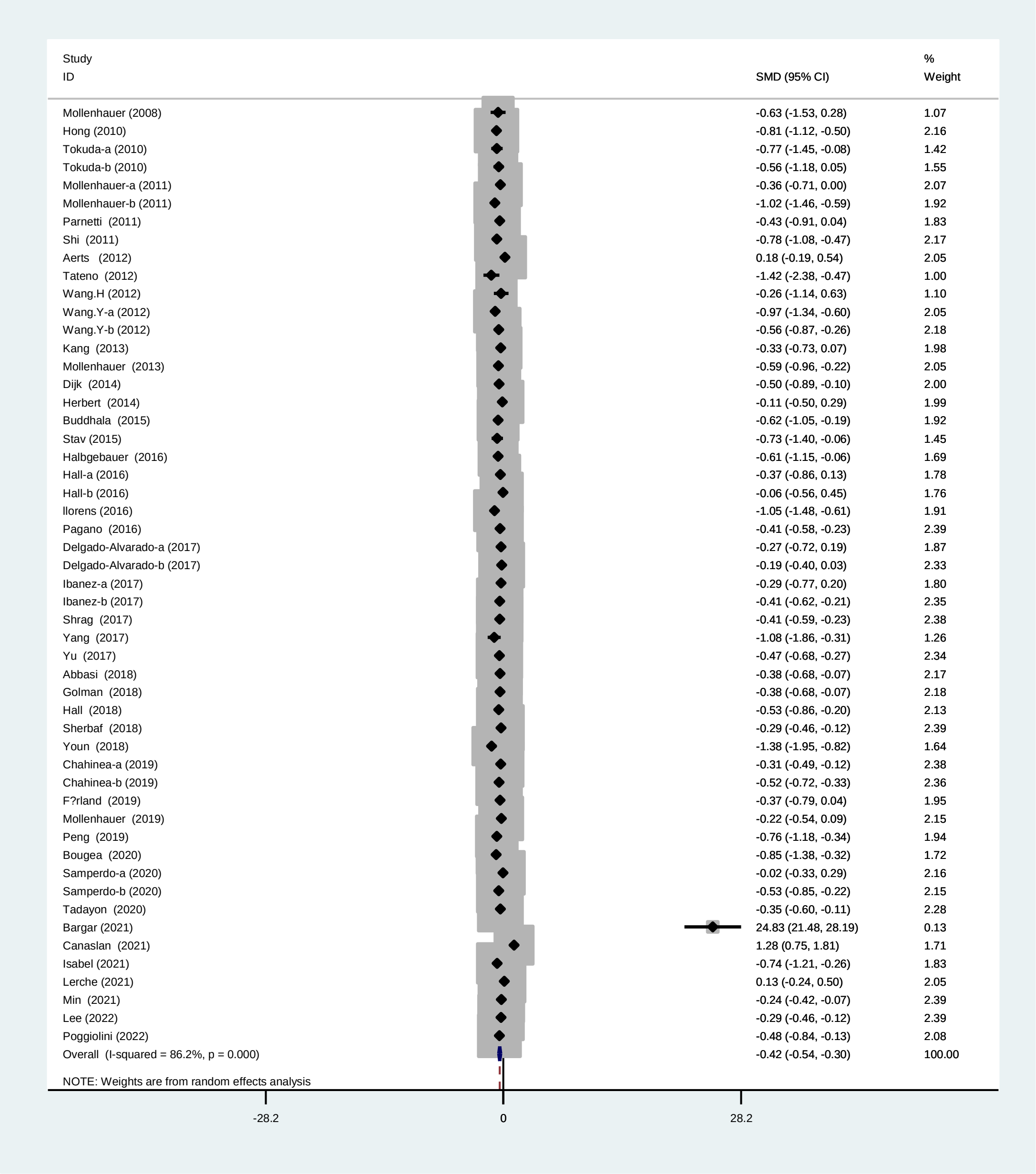

(B) PD&HC group

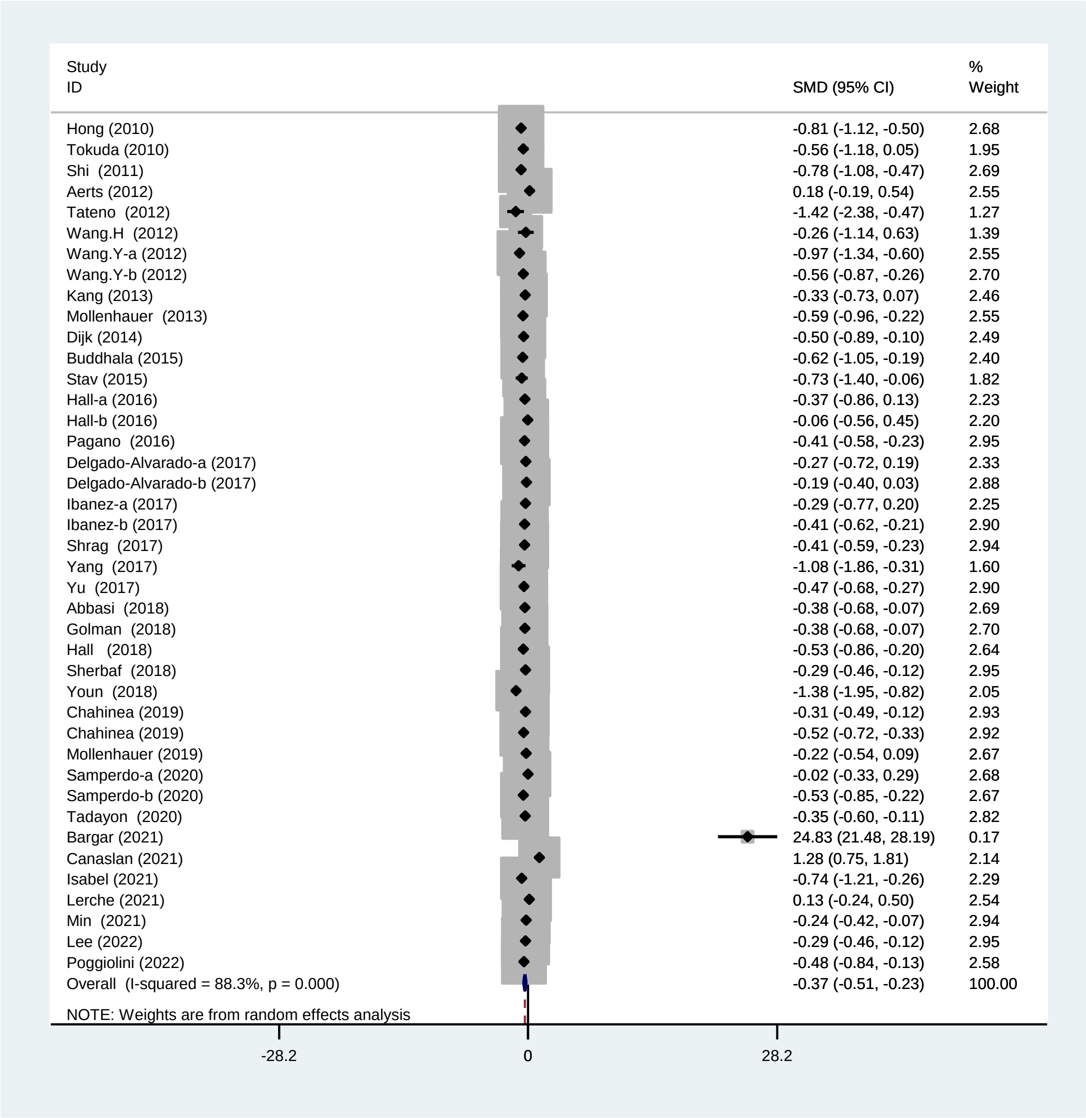
Study
ID
SMD (95% CI)
%
Weight
Hong (2010) -0.81 (-1.12, -0.50) 2.68
Tokuda (2010) -0.56 (-1.18, 0.05) 1.95
Shi (2011) -0.78 (-1.08, -0.47) 2.69
Aerts (2012) 0.18 (-0.19, 0.54) 2.55
Tateno (2012) -1.42 (-2.38, -0.47) 1.27
Wang.H (2012) -0.26 (-1.14, 0.63) 1.39
Wang.Y-a (2012) -0.97 (-1.34, -0.60) 2.55
Wang.Y-b (2012) -0.56 (-0.87, -0.26) 2.70
Kang (2013) -0.33 (-0.73, 0.07) 2.46
Mollenhauer (2013) -0.59 (-0.96, -0.22) 2.55
Dijk (2014) -0.50 (-0.89, -0.10) 2.49
Buddhala (2015) -0.62 (-1.05, -0.19) 2.40
Stav (2015) -0.73 (-1.40, -0.06) 1.82
Hall-a (2016) -0.37 (-0.86, 0.13) 2.23
Hall-b (2016) -0.06 (-0.56, 0.45) 2.20
Pagano (2016) -0.41 (-0.58, -0.23) 2.95
Delgado-Alvarado-a (2017) -0.27 (-0.72, 0.19) 2.33
Delgado-Alvarado-b (2017) -0.19 (-0.40, 0.03) 2.88
Ibanez-a (2017) -0.29 (-0.77, 0.20) 2.25
Ibanez-b (2017) -0.41 (-0.62, -0.21) 2.90
Shrag (2017) -0.41 (-0.59, -0.23) 2.94
Yang (2017) -1.08 (-1.86, -0.31) 1.60
Yu (2017) -0.47 (-0.68, -0.27) 2.90
Abbasi (2018) -0.38 (-0.68, -0.07) 2.69
Golman (2018) -0.38 (-0.68, -0.07) 2.70
Hall (2018) -0.53 (-0.86, -0.20) 2.64
Sherbaf (2018) -0.29 (-0.46, -0.12) 2.95
Youn (2018) -1.38 (-1.95, -0.82) 2.05
Chahinea (2019) -0.31 (-0.49, -0.12) 2.93
Chahinea (2019) -0.52 (-0.72, -0.33) 2.92
Mollenhauer (2019) -0.22 (-0.54, 0.09) 2.67
Samperdo-a (2020) -0.02 (-0.33, 0.29) 2.68
Samperdo-b (2020) -0.53 (-0.85, -0.22) 2.67
Tadayon (2020) -0.35 (-0.60, -0.11) 2.82
Bargar (2021) 24.83 (21.48, 28.19) 0.17
Canaslan (2021) 1.28 (0.75, 1.81) 2.14
Isabel (2021) -0.74 (-1.21, -0.26) 2.29
Lerche (2021) 0.13 (-0.24, 0.50) 2.54
Min (2021) -0.24 (-0.42, -0.07) 2.94
Lee (2022) -0.29 (-0.46, -0.12) 2.95
Poggiolini (2022) -0.48 (-0.84, -0.13) 2.58
Overall (I-squared = 88.3%, p = 0.000) -0.37 (-0.51, -0.23) 100.00
NOTE: Weights are from random effects analysis
-28.2
0
28.2

(C) PD&OND group

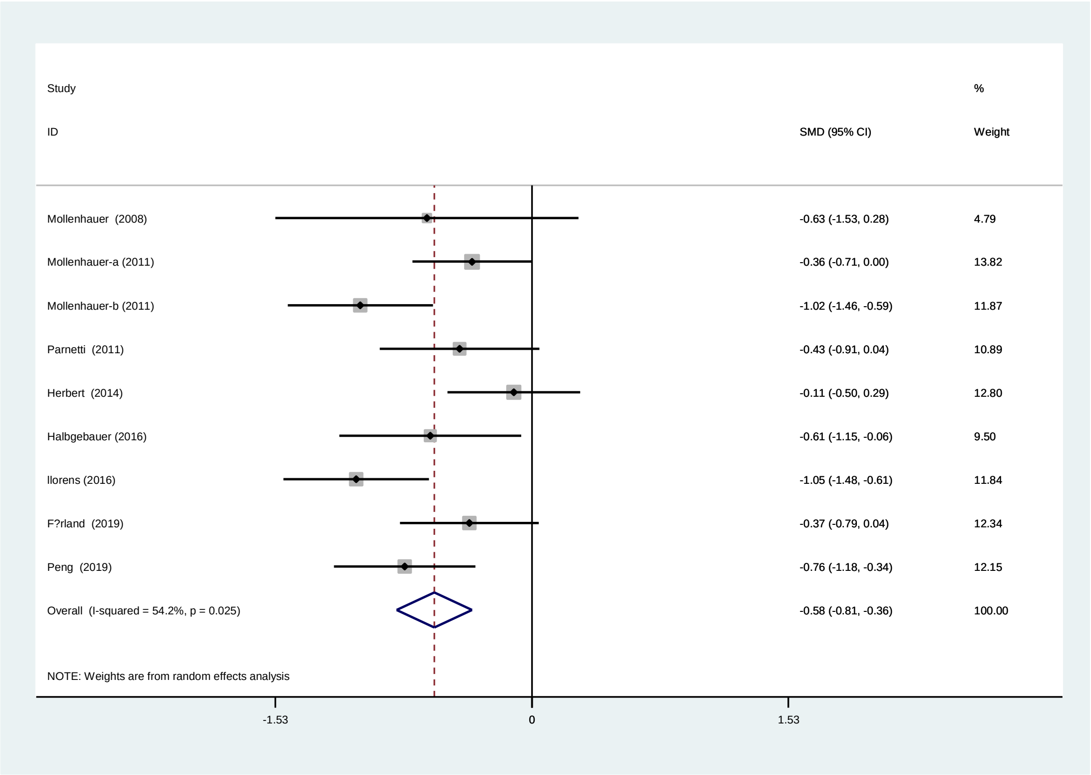


(D) PD&MSA group

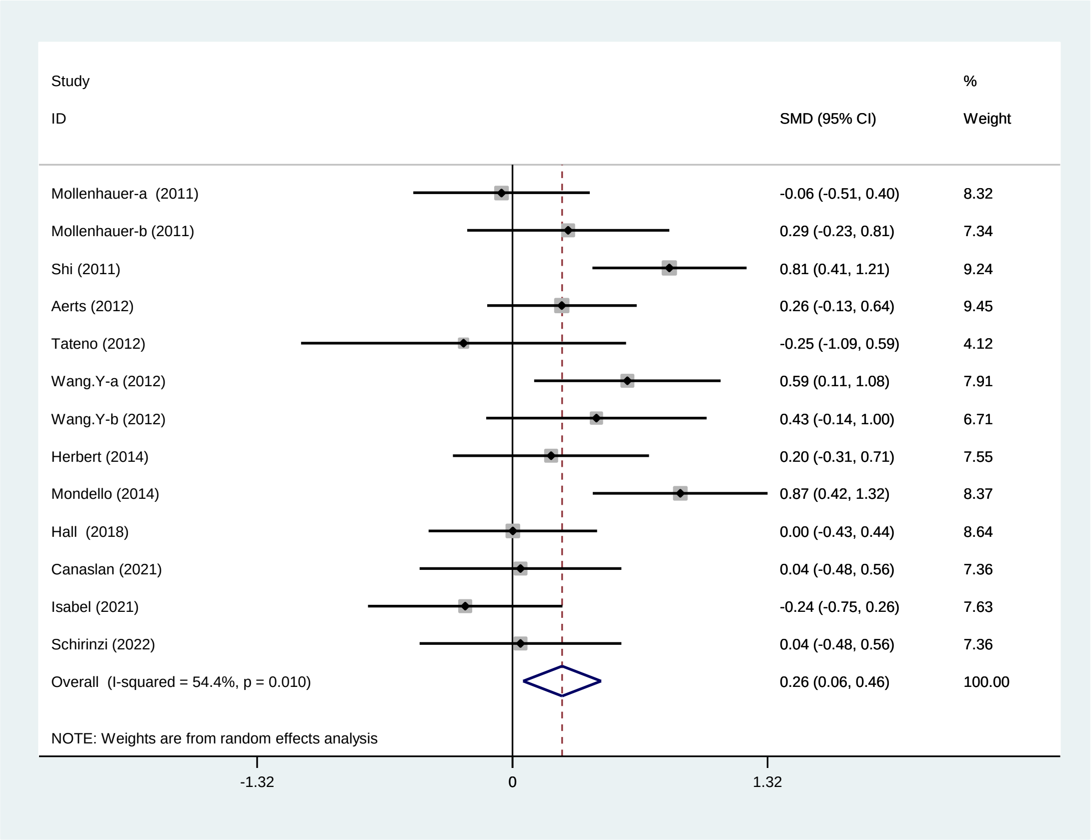

(E) PD&PSP

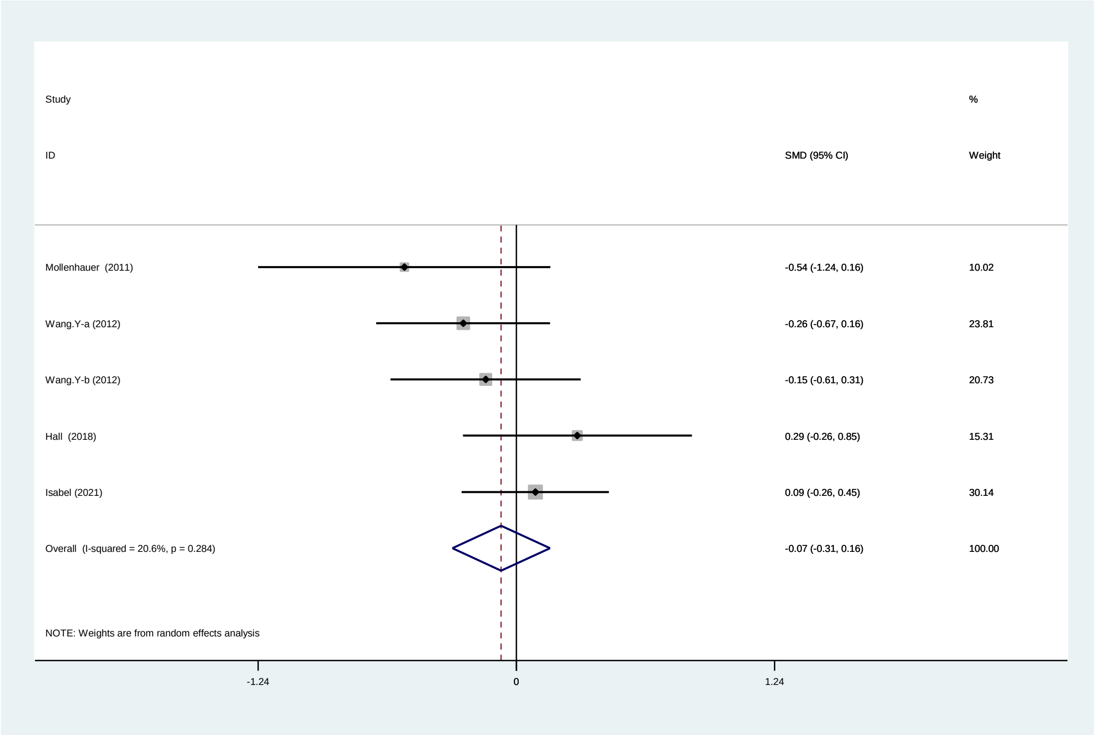


(F) PD&DLB

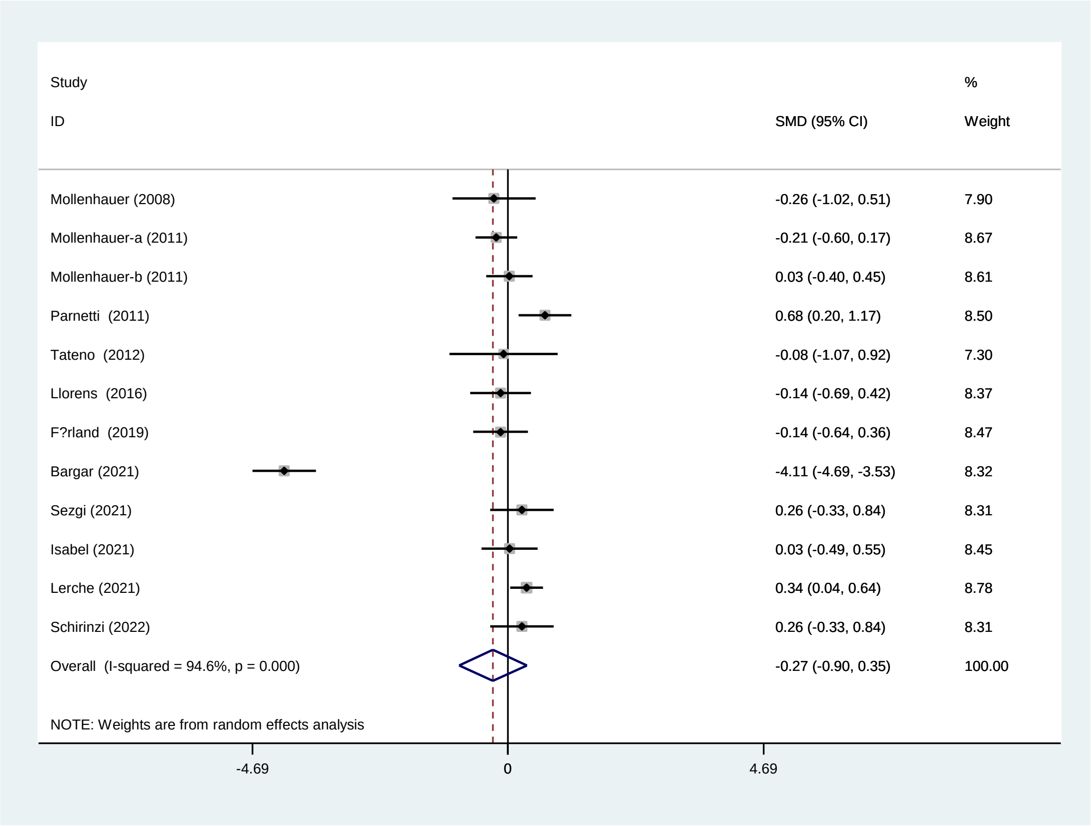

**Supplementary material 11**. Forest plot for o-α-syn and p-α-syn in PD& Control group. (A) o-α-syn in PD &Control group; (B) o-α-syn in PD&HC group; (C) o-α-syn in PD&OND group; (D) p-α-syn in PD &Control group; effect size (risk ratio) calculated using a random effect model. ES, effective size; CI, confidence intervals; SMD, standardized mean difference.

(A) o-α-syn in PD& Control group

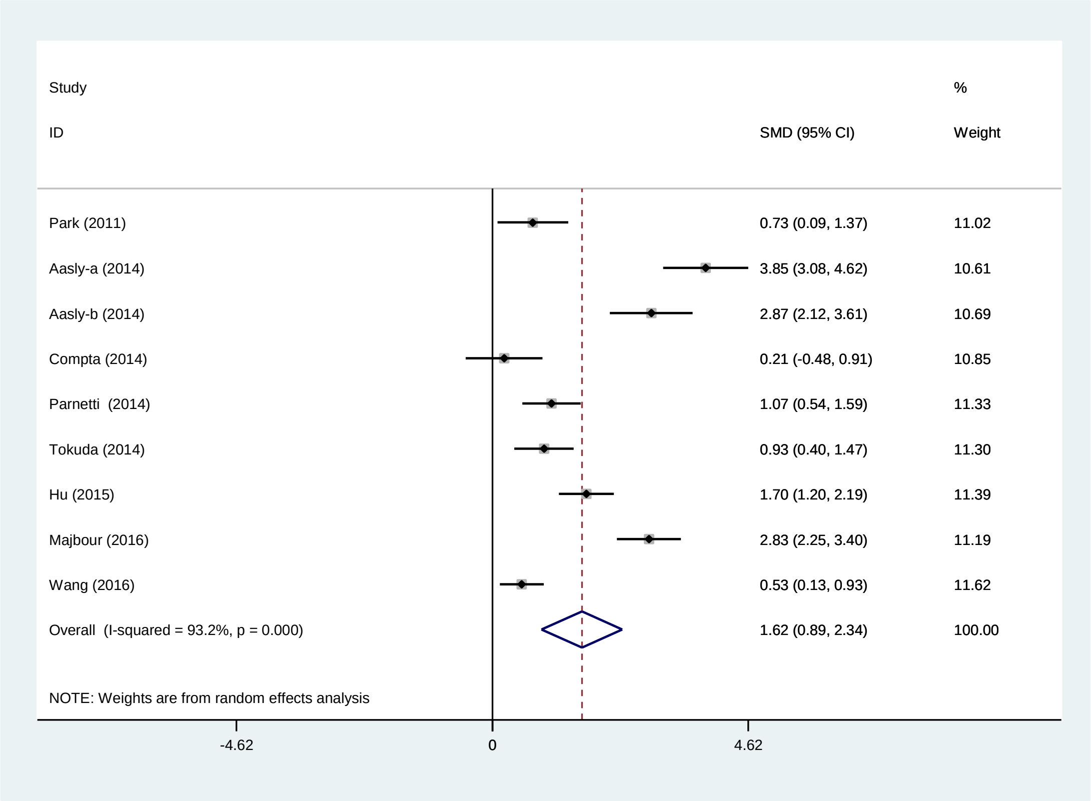


(B) o-α-syn in PD&HC group

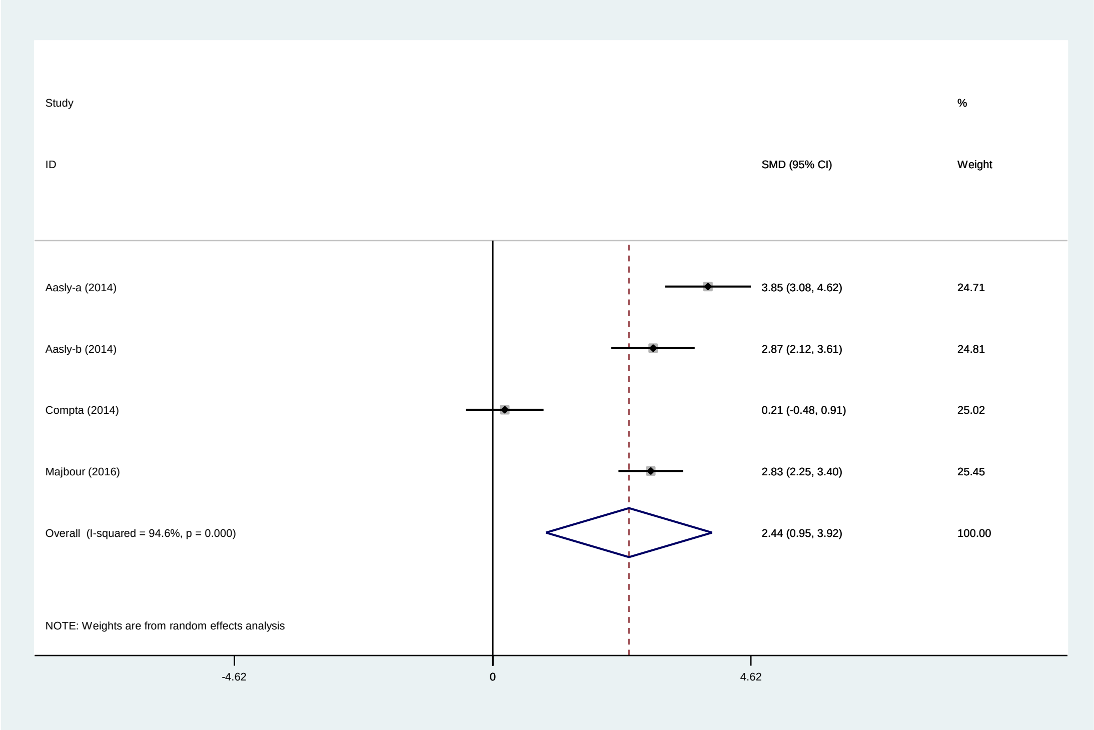

(C) o-α-syn in PD&OND group

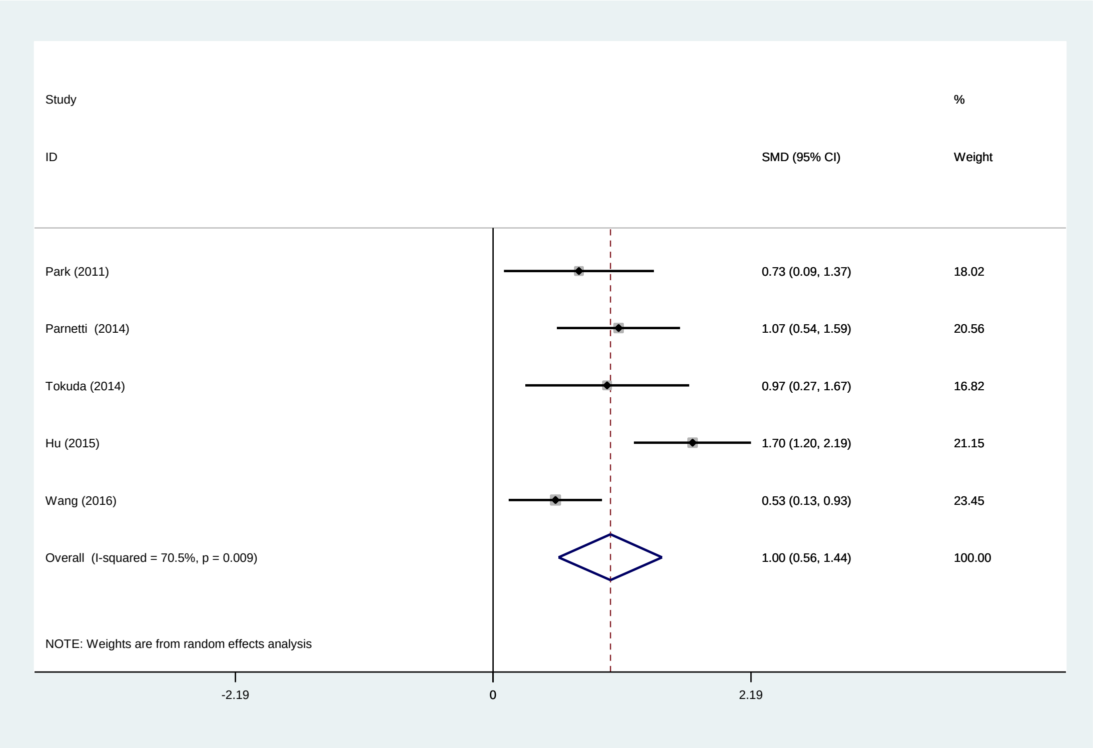


(D) p-α-syn in PD &Control group

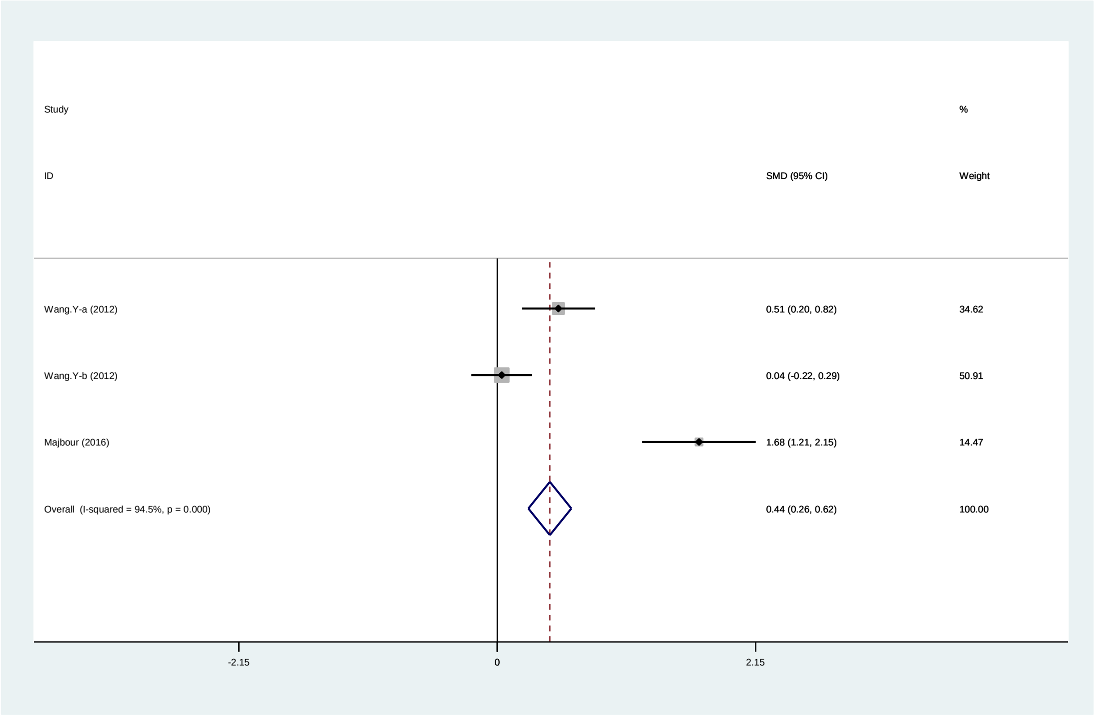

**Supplementary Table 12.** Summary of subgroup analysis results of assay type, age and disease process. (A) Subgroup analysis results of α-syn; (B) Subgroup analysis results of NFL; (C) Subgroup analysis results of p-tau; (D) Subgroup analysis results of t-tau; (E) Subgroup analysis results of Aβ42;

**(A)** Subgroup analysis results of α-syn;

| t-α-syn | | | | | |
|---|---|---|---|---|---|
| **Moderator** | **Subgroup** | **N** | **S** | **SMD (95%CI)** | **$I^2$** |
| **1.Assay type** | ELISA | 6501 | 46 | -0.413(-0.498 to -0.327) | 66.8% |
| | RT-QuIC | 3762 | 2 | 12.118(-12.691 to 36.927) | 99.5% |
| | Luminex | 396 | 4 | -0.668(-0.915 to -0.421) | 52.1% |
| **2.Age** | High | 1679 | 19 | -0.317(-0.690 to 0.057) | 93.0% |
| | Low | 3935 | 21 | -0.428(-0.522 to -0.334) | 59.2% |
| **3.Disease process** | De novo | 1453 | 10 | -0.429(-0.569 to -0.289) | 56.4% |
| | Non-de novo | 5551 | 35 | -0.411(-0.569 to -0.254) | 88.7% |

**(B)** Subgroup analysis results of NFL;

| NFL | | | | | |
|---|---|---|---|---|---|
| **Moderator** | **Subgroup** | **N** | **S** | **SMD (95%CI)** | **$I^2$** |
| **1.Age** | High | 587 | 6 | 0.269(0.025 to 0.513) | 47.8% |
| | Low | 185 | 4 | -0.010(-0.463 to 0.443) | 77.0% |
| **2.Disease process** | De novo | 1191 | 8 | 0.162(-0.030 to 0.354) | 0.0% |
| | Non-de novo | 568 | 7 | 0.327(-0.051 to 0.705) | 82.4% |

**(C)** Subgroup analysis results of p-tau;

| p-tau | | | | | |
|---|---|---|---|---|---|
| **Moderator** | **Subgroup** | **N** | **S** | **SMD (95%CI)** | **$I^2$** |
| **1. Assay type** | ELISA | 6274 | 46 | -0.301 (-0.380 to -0.222) | 61.4% |
| | Luminex | 396 | 3 | -0.461(-0.745 to -0.178) | 32.8% |
| | Others* | 149 | 2 | -0.139(-0.384 to 0.107) | 0.0% |
| **2.Age** | High | 1469 | 19 | -0.249(-0.462 to -0.037) | 79.1% |
| | Low | 4392 | 28 | -0.316(-0.372 to -0.259) | 10.3% |
| **3.Disease process** | De novo | 1191 | 8 | -0.520(-0.271 to -0.368) | 51.1% |
| | Non-de novo | 5445 | 39 | -0.249(-0.329 to -0.170) | 56.4% |

**(D)** Subgroup analysis results of t-tau;

| t-tau | | | | | |
|---|---|---|---|---|---|
| **Moderator** | **Subgroup** | **N** | **S** | **SMD (95%CI)** | **I²** |
| **1. Assay type** | ELISA | 5919 | 57 | -0.256(-0.345 to -0.166) | 68.3% |
| | Luminex | 396 | 2 | -0.406(-0.615 to -0.197) | 0.0% |
| | Others* | 177 | 3 | -0.44(-0.506 to -0.291) | 0.0% |
| **2.Age** | High | 1951 | 22 | -0.265(-0.389 to 0.018) | 28.7% |
| | Low | 6925 | 56 | -0.258(-0.325 to -0.190) | 53.8% |
| **3.Disease process** | De novo | 1265 | 7 | -0.391(-0.504 to -0.278) | 21.4% |
| | Non-de novo | 6103 | 49 | -0.287(-0.387 to -0.187) | 70.4% |

**(E)** Subgroup analysis results of Aβ42;

| Aβ42 | | | | | |
|---|---|---|---|---|---|
| **Moderator** | **Subgroup** | **N** | **S** | **SMD (95%CI)** | **I²** |
| **1. Assay type** | ELISA | 6560 | 57 | -0.228(-0.303 to -0.153) | 58.7% |
| | Luminex | 396 | 2 | -0.405(-0.614 to -0.196) | 0.0% |
| | Others* | 177 | 3 | -0.328(-0.546 to -0.109) | 0.0% |
| **2.Age** | High | 2005 | 20 | -0.184(-0.304 to -0.065) | 53.6% |
| | Low | 6431 | 51 | -0.247(-0.321 to -0.173) | 59.3% |
| **3.Disease process** | De novo | 1206 | 8 | -0.279(-0.396 to -0.162) | 24.7% |
| | Non-de novo | 5720 | 44 | -0.237(-0.318 to -0.156) | 59.5% |

Subgroups of assay type, age and disease process based on tertiles of study averages. ES, effect size; CI, confidence intervals; S, number of included studies; N, number of patients; α-synuclein, α-syn; NFL, neurofilament light-chain protein; Aβ42, the 42-amino-acid form of Aβ; t-tau, total tau; p-tau, phosphorylated tau; *, Innotest Phospho-Tau (181) -assay, Fujirebio GmbH INNOTEST assay, (CSF) lactate concentration and Quantitative analysis of cerebrospinal fluid.
